# Metagenomic Insights Into the Microbial Community and Nutrient Cycling in the Western Subarctic Pacific Ocean

**DOI:** 10.3389/fmicb.2018.00623

**Published:** 2018-04-04

**Authors:** Yingdong Li, Hongmei Jing, Xiaomin Xia, Shunyan Cheung, Koji Suzuki, Hongbin Liu

**Affiliations:** ^1^Division of Life Science, The Hong Kong University of Science and Technology, Kowloon, Hong Kong; ^2^CAS Key Laboratory for Experimental Study Under Deep-Sea Extreme Conditions, Institute of Deep-Sea Science and Engineering, Chinese Academy of Sciences, Sanya, China; ^3^Faculty of Environmental Earth Science, Hokkaido University, Sapporo, Japan

**Keywords:** microbial community, niche differentiation, nutrient cycle, metagenome, metabolism, subarctic Pacific Ocean

## Abstract

The composition and metabolic functions of prokaryotic communities in the western subarctic Pacific (WSP), where strong mixing of waters from the Sea of Okhotsk and the East Kamchatka Current result in transfer to the Oyashio Current, were investigated using a shotgun metagenome sequencing approach. Functional metabolic genes related to nutrient cycling of nitrogen, sulfur, carbohydrates, iron and amino acids were differently distributed between the surface and deep waters of the WSP. Genes related to nitrogen metabolism were mainly found in deep waters, where *Thaumarchaeaota, Sphingomonadales*, and *Pseudomonadales* were closely associated and performing important roles in ammonia oxidation, assimilatory nitrate reduction, and dissimilatory nitrate reduction processes, respectively. In addition, orders affiliated to *Spingobacteria* and *Alphaproteobacteria* were crucial for sulfate reduction and abundant at 3000 m, whereas orders affiliated to *Gammaproteobacteria*, which harbored the most sulfate reduction genes, were abundant at 1000 m. Additionally, when compared with the East Kamchatka Current, the prokaryotes in the Oyashio Current were likely to consume more energy for synthesizing cellular components. Also, genes encoding iron transport and siderophore biosynthesis proteins were in low abundance, indicating that the iron was not a limiting factor in the Oyashio current. In contrast, in the East Kamchatka Current, prokaryotes were more likely to directly utilize the amino acids and absorb iron from the environment. Overall, our data indicated that the transformation from the East Kamchatka Current to the Oyashio Current reshapes not only the composition of microbial community, but also the function of the metabolic processes. These results extended our knowledge of the microbial composition and potential metabolism in the WSP.

## Introduction

Microbes in marine ecosystems are the most important drivers of biogeochemical cycling on a global scale (Azam et al., 1983), since they are responsible for the re-mineralization of organic matter and the transfer of both nutrients and energy to the higher trophic levels in the ocean (Nealson, 1997; Mason et al., 2009). In diverse oceanic environments, microbes in individual taxonomic units might evolve or become extinct, but the essential metabolic genes remain unperturbed (Falkowski et al., 2008). Therefore, in order to gain a better understanding of the basic mechanisms that control microbial processes and interactions *in situ*, one of the main priorities of microbial ecologists today is to identify which ecological process (such as carbon, nitrogen, or sulfur cycling) is regulated by a particular microbial community (Prosser, 2012; Llorens-Marès et al., 2015). The recent development of high throughput sequencing of the metagenome now enables researchers to investigate and compare the community structure of microbial assemblages from various environments, and to study their metabolic potential (Thompson et al., 2017).

The metagenomics approach has previously been applied to investigate the diversity and abundance of genes associated with nitrogen, sulfur, carbon and phosphorous metabolism, in bodies of water such as the vertical water column in the North Pacific Subtropical Gyre (DeLong et al., 2006), the central Pacific Ocean (Hewson et al., 2009), the California Current (Allen et al., 2012), the Dead Sea (Bodaker et al., 2010), the Mediterranean deep-sea brines (Smedile et al., 2013), the Mariana Trench (Nunoura et al., 2015) and the southwestern Atlantic Ocean (Junior et al., 2015). However, until now, a general lack of genomic information in the western subarctic Pacific (WSP) Ocean has hindered our understanding of the adaptation of microbes to the subarctic environment with near-freezing temperatures and diverse marine geography.

The Sea of Okhotsk is characterized by low temperature and segregated by the Kuril Island chain from the North Pacific Ocean. The Kruzenshtern and Bussol Straits, two major passages in the Kuril Island chain, enable the exchange of flow between the Sea of Okhotsk and the North Pacific Ocean (Kida and Qiu, 2013). Furthermore, the strong mixing of water of the East Kamchatka Current (one branch of the Bering slope current), with water from the Okhotsk Sea at the Bussol Strait, plays a major role in the transformation of the East Kamchatka Current to the Oyashio Current (Ohtani, 1991). In addition, the Sea of Okhotsk has very high biological production among the world’s oceans, especially along its continental shelf where a series of successive large diatom blooms occur in early summer (Orlova et al., 2004; Suzuki et al., 2011), which were in part supported by the discharge of iron (Fe) and nutrient-rich water from the Amur River (Nishioka et al., 2007). With all these unique bio-geographic conditions and little information about the composition and metabolism potential of the microbial community in the WSP, we hypothesize that (1) the microbial community and metabolic function in WSP cold water may be different from other marine regions; (2) in the open ocean of the WSP, metabolic function of microbial communities may be different in the surface and deep waters; (3) the mixing of water from the Sea of Okhotsk with the East Kamchatka Current at Bussol Strait may shape a unique microbial community and suite of metabolic functions.

Here, we carried out a metagenomics study based on samples collected from five stations in the WSP region. Three of the stations were located in the Bussol Strait and the Sea of Okhotsk, and one sampling station was in the East Kamchatka Current. In addition, the mesopelagic and bathypelagic water (i.e., 1000 and 3000 m, respectively) were collected at a pelagic station. This sampling strategy enabled us to compare the prokaryotic community structure and functional genes in waters subjected to different ocean currents as well as in the upper and deep layers of the WSP. Our work identified the mixing of water in the WSP dramatically changed the distribution of prokaryotes encoding genes in carbohydrate metabolism, amino acid metabolism, and iron transport. It also revealed that several microbial taxa, were more abundant in deep waters and tightly related with nitrogen metabolism. Furthermore, by comparing with other related studies, the unique features of the WSP were revealed and discussed.

## Materials and Methods

### Sample Collection, Genomic DNA Extraction and Sequencing

**FIGURE 1:**
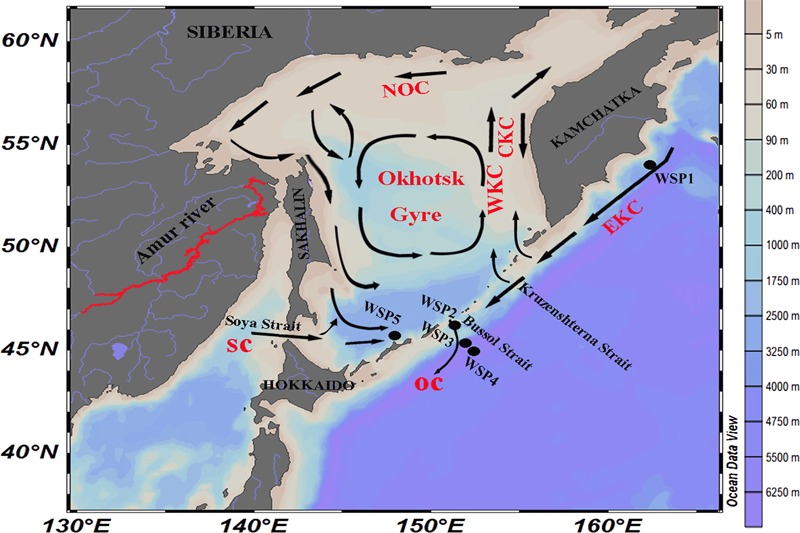
Location of the sampling stations around the Sea of Okhotsk and North Western Subarctic Pacific Ocean. NOC, North Okhotsk Current; WKC, West Kamchatka Current; EKC, East Kamchatka Current; CKC, Compensation Kamchatka Current; SC, Soya Current; and OC, Oyashio current.

Samples were collected from 5 stations located in the western sub-arctic Pacific Ocean aboard the R/V *Professor Multanovskyi* (FERRHI, Russia) in June 2014 (**Figure 1**). Approximately 40 l of seawater were collected from the surface of Stn. WSP1, WSP2, WSP3, and WSP5. In addition, for comparison purposes, water from depths of 1000 and 3000 m were collected at Stn. WSP4. Water was collected using a conductivity-temperature-depth rosette system (CTD, General Oceanics, Miami, FL, United States) with X-Niskin bottles (General Oceanics, Miami, FL, United States); it was then pre-filtered through 3.0 μm filters, and then filtered onto 0.22 μm pore-size polycarbonate membranes (both 47 mm diameter, EMD Millipore, Billerica, MA, United States). All the filters were then flash frozen and stored at -80°C until DNA extraction could be conducted on land. Various *in situ* environmental parameters (i.e., temperature, salinity, depth, and dissolved oxygen) were recorded with the CTD. Samples for measuring inorganic nutrients (i.e., nitrate, nitrite, ammonia, phosphate, and silicate) were collected in acrylic tubes and analyzed in the onboard laboratory. The concentrations of the nutrients were measured with an auto-analyzer (QuAAtro, BLTEC. Co., Ltd.), which was calibrated with certified seawater nutrient reference material (RM; KANSO).

Genomic DNA was extracted from the 0.22 μm polycarbonate filters with a PureLink Genomic DNA Kit (Invitrogen, Thermo Fisher Scientific Corp., Carlsbad, CA, United States), eluted into 100 μl Tris-EDTA (TE) buffer and stored at -80°C. The shotgun sequencing of extracted DNA was first sheared to 250 bp using a focused ultrasonicator (Covaris, Woburn, MA, United States), and then performed with an Illumina HiSeq2500 PE 150 and the HiSeq Cluster Kit v4 (San Diego, CA, United States) by the South Gene Company (Shanghai, China).

### Bioinformatics Analysis

#### Sequence Processing

The sequencing data were first assigned to each sample according to their barcode. The merged short sequences were then trimmed to remove the barcode and high quality sequences (length > 140 bp, without ambiguous base “N,” and average base quality > 20) were selected by the FASTX-Toolkit for further analysis (Pearson et al., 1997). All the taxonomic and functional annotations were based exclusively on the quality reads. The number of extracted and qualified sequence, average length and average GC content of each sample are listed in Supplementary Table S2. The quality sequences are available from NCBI SRA dataset under the bioproject PRJNA398459.

#### Prokaryotic Taxonomic Assignment and Analysis

The short paired-end quality reads were first merged and converted into one fasta file, then Metaxa2 (Bengtsson-Palme et al., 2015) was used for SSU rRNAs screening. The obtained SSU rRNAs reads were used to align the 16S rRNA reference database^1^ (Quast et al., 2012) in QIIME for further taxonomic assessment. The annotation and classification system we used here follows the Silva database (version 123). Following the analysis pipe-line described in former studies (Caporaso et al., 2010), the retrieved reads were analyzed, and the alpha and beta diversity of prokaryotes was evaluated via QIIME 1.9.1. A filtered OTUs table at 0.1% abundance of each sample was generated with QIIME 1.9.1, and the summarize_taxa.py script was used to treat the OTUs table into relative abundances for Lda (linear discriminant analysis) effective size (LEfSe) analysis, which was then typically used to compare taxonomic units between surface and deep samples (Segata et al., 2011). Furthermore, the SIMPER (Similarity Percentage) statistical method in Past3 software (Hammer et al., 2001) was used to select the taxonomic units that contribute most to the differences between WSP1 and other surface samples, and the top 50 taxonomic units of each comparison (WSP1 to WSP2, WSP1 to WSP3, and WSP1 to WSP5) were selected for visualization.

#### Metagenome Assembly and Functional Assignment

The IDBA-UD (Peng et al., 2012) algorithm was applied to achieve an iterative *de novo* assembly using a kmer size iterated from 20 to 100 with a step size of 10 and a pre-correction step by a kmer size of 60. The open reading frames (ORFs) of prokaryotic organisms were identified by the Prodigal software (Peng et al., 2012) with an ORF length set at a minimum of 400 bp for predicted genes. The predicted genes were used to hit the Kyoto Encyclopedia of Genes and Genomes (KEGG) (Kanehisa et al., 2007) and non-redundant protein (NR) database via the Diamond software (Buchfink et al., 2015) with blastp, -k 1 and -e 10^-10^ parameters to select the best annotation result. Genes encoding carbohydrate metabolizing enzymes were identified by using the predicted genes to against the Carbohydrate-Active enZYmes (CAZY) public database (Cantarel et al., 2009) with the same filtration mechanism; the CAZY database was localized into the lab server by using the source from the web server dbCAN (Yin et al., 2012). In each sample, the raw reads were used to map back to the predicted genes by the software, Bowtie 2.2.9 (Langmead and Salzberg, 2012) to get the accurate abundance of each gene. The blast result of NR database was further imported into MEGAN 6 software (Buchfink et al., 2015). After loading the accession mapping file provided by the MEGAN software website, the taxonomy of each gene’s microbial affiliation was extracted by the export function in MEGAN 6 software (Beier et al., 2017). In order to distinguish the genotypes of each nitrogen metabolism-related gene, positive genes (genes longer than 500 bases) in each sample were first selected and imported into QIIME software. Then the pick_otus.py with -s 0.97 parameter was used to cluster the selected genes into OTUs. Finally, the representative sequence of each OTU unit was picked out by pick_rep_set.py script in QIIME. The maximum likelihood tree of these representative sequences was constructed using MEGA 6.06 accompanied by the top hit reference sequences downloaded from NCBI. Furthermore, among these genes the classification of *amoA* was based on the description by Beman et al. (2008).

### Statistical Analysis and Network Analysis

The distribution patterns of the microbial communities were analyzed through hierarchical clustering based on the Bray-Curtis similarity index, using PRIMER 5 (Plymouth Marine Laboratory, West Hoe, Plymouth, United Kingdom), and hierarchical clustering analysis in SPSS software was used to verify the cluster result of PRIMER. The SIMPER statistical method in Past3 software was used to select the functional categories and microbial taxa that contribute most to the differences between groups.

Co-occurrence network analyses were conducted with SparCC (Friedman and Alm, 2012) using the Spearman method. In order to achieve the median correlation of each pairwise microbial taxa, ten iterations were conducted, and the statistical significance of the correlations was calculated by bootstrapping with 500 iterations. The Benjamini–Hochberg method was used for the correction of multiple-testing of the *p*-values, and correlations with *p-*value < 0.01 and *R*-value ≤ 0.6 and ≥-0.6 were sorted and visualized using the ‘corrplot’ package in R.

## Results

### Hydrographic Conditions

Among the various sampling stations (**Figure 1**), WSP1 was located in the Pacific side of Kamchatka Peninsula. It exhibits the lowest surface water temperature (1.8°C) and nutrient level (NO_2_ + NO_3_: 0.1 μmol/kg; NH_4_: <0.2 μmol/kg; PO_4_^-^: 0.13 μmol/kg; Si: <0.5 μmol/kg) (Supplementary Table S1). WSP2 was located adjacent to the Bussol Strait; it also had a relatively low surface water temperature (3.4°C), but a high nutrient level (NO_2_ + NO_3_: 14.6 μmol/kg; NH_4_: <0.2 μmol/kg; PO_4_^-^: 0.99 μmol/kg; Si: 26.4 μmol/kg). WSP3 and WSP5 were located further into the Western Subarctic Gyre, and in the deep basin of the Sea of Okhotsk, respectively, and they both exhibited hydrographic conditions similar to WSP2. Also, it was noticeable that the concentrations of nitrate and nitrite were markedly higher at WSP2 and WSP3 (13.07–14.6 μmol kg^-1^) than at WSP1 (0.1 μmol kg^-1^).

### The Relationship of the Prokaryotic Communities

After trim and assembly, a total of 681,578 contigs (of >500 bp) were obtained, ranging from 78,014 to 136,635 contigs in each sample (Supplementary Table S2). In total, 3,476 OTUs could be assigned at a 97% sequence similarity threshold, and the 16S rRNA gene reads accounted for 0.473, 0.493, 0.325, 0.664, 0.662, and 0.534% in WSP1, WSP2, WSP3, WSP4_1000m, WSP4_3000m and WSP5, respectively.

**FIGURE 2:**
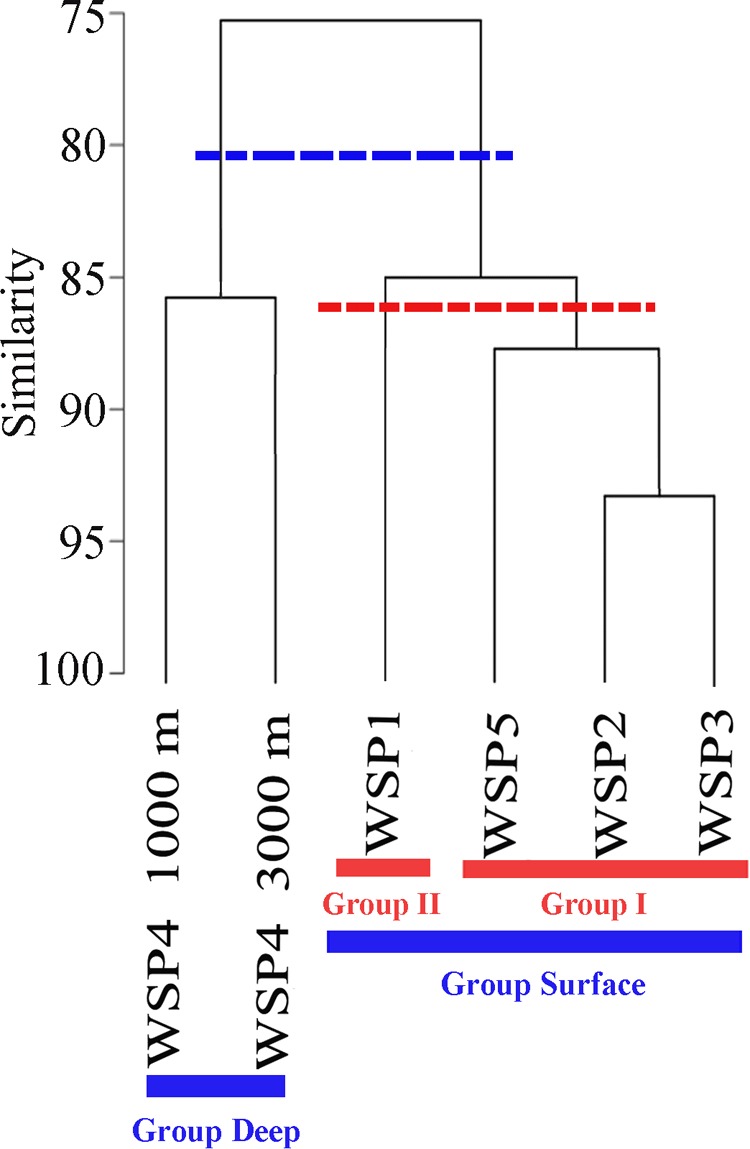
Community similarities among the samples based on the UPGMA clustering with the abundance of 16S OTUs. The cluster dendrograms were constructed based on Bray–Curtis similarity using PRIMER5.

The relationship among the prokaryotic communities was investigated via Bray–Curtis similarity and hierarchical cluster analysis. At the similarity cut-off value of 80% (**Figure 2**), the samples could be separated into two groups: surface (WSP1, WSP2, WSP3, and WSP5) and deep (WSP4_1000m and WSP4_3000m). By using the hierarchical clustering method to conduct two groups clustering, the same clustering result was generated with the Kaiser-Meyer-Olkin (KMO) test value > 0.85 and Bartlett’s test value < 0.01, which means that the data was suitable for this cluster method and the result was relatively accurate (Supplementary Table S3). After clustering, the components in each group could explain over 80% of the information in each sample (Supplementary Table S4). Between the surface samples, at the similarity cut-off value of 87%, the samples can be separated into two different groups. The same cluster result was generated when we used the hierarchical cluster method to analyze the surface samples, and the Kaiser-Meyer-Olkin (KMO) and Bartlett’s test showed similar results with the former two groups’ clustering (Supplementary Table S5). Also, over 80% of the information within these surface samples was well covered in this clustering method (Supplementary Table S6).

### Prokaryotic Community Composition

**FIGURE 3:**
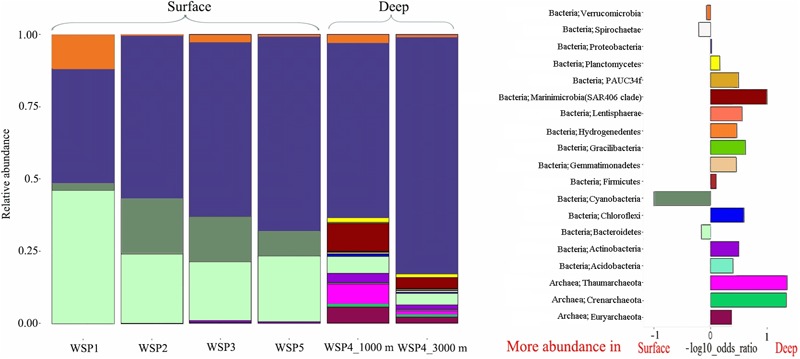
The microbial community composition at the phylum level **(left)**, and a differential analysis between the surface and deep-water groups **(right)**. For the differential analysis, values are the base-10 logarithm of the odds ratio, and the positive and negative values indicate that the taxa are more abundant in the deep and surface waters, respectively.

The microbial diversity in deep waters was much higher than that in the surface waters at all the stations (**Figure 3**). *Proteobacteria* was predominant throughout the WSP (55.3%, on average), with the highest relative abundance at WSP4 at 3000 m (75.6%). Two other common phyla, *Verrucomicrobia* and *Bacteroidetes*, were also widely distributed at all the stations. The relative abundance of *Verrucomicrobia* ranged from 0.3% to 11.7% in our samples, with the highest abundance found at WSP1 (11.7%). *Bacteroidetes* showed a higher abundance in the surface waters relative to deep waters, and its proportion (45.1%) at WSP1 was higher than *Proteobacteria*. *Cyanobacteria* was also mainly distributed in the surface samples, with higher abundance at WSP2 and WSP3 (16.7%, on average) than WSP1 (2.5%). Two widely distributed Archaea phylum, *Thaumarchaeota*, and *Euryarchaeota*, were abundant in the deep sea samples, accounting for 6.4 and 3.3% at 1000 m, respectively, and 1.4 and 1.3% at 3000 m, respectively.

**FIGURE 4:**
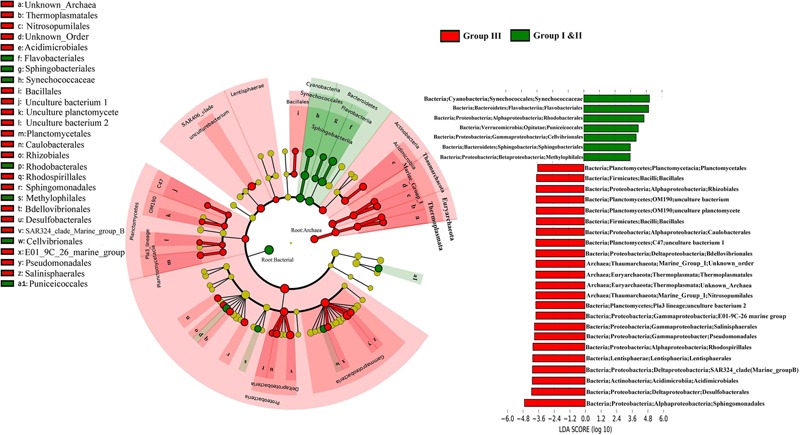
The cladogram indicates the phylogenetic distribution of the microbial lineages in the surface and deep water. The average abundance of microbial taxa in two groups was used in the differential analysis. The LDA score of the taxa representing the odds of their distribution between surface and deep. The phylum, class and order are listed in order from the inside to the outside of each cladogram.

The Lda analysis showed a vertical differentiation in the microbial composition in the WSP (**Figure 4**). Although bacteria were more prevalent in the surface waters (Group I and II), and Archaea were more common in deep waters (Group III), the distribution of microorganisms of different taxonomic levels still existed when comparing the surface and deep-water samples. For example, *Deltaproteobacteria* and *Gammaproteobacteria* were more abundant in Group III. The prevalence of *Deltaproteobacter* was reflected by the increase of *Desulfobacterales* (-4.1 LDA), *SAR324_clade_marine_group_B* (-3.8 LDA), and *Bdellovibrionales* (-3.7 LDA); whereas that of *Gammaproteobacteria* was reflected by the increase of *E01_9C_26 Marine group* (-3.7 LDA), *Pseudomonadales* (-3.8 LDA), and *Salinisphaerales* (-3.8 LDA). Within the Archaea, *Thermoplasmatales* (assigned to *Euryarchaeota Thermoplasmata*), and *Nitrosopumilales* (assigned to *Thaumarchaeota Marine Group I*), were both significantly enriched in Group III with a similar LDA score of around -3.6. In addition, *Synechococcaceae* and *Flavobacteriales*, which are affiliated to *Cyanobacteria* and *Bacteroidetes*, respectively, were the two most surface abundant taxa, with LDA scores of 4.83 and 4.82, respectively.

After pairwise comparisons between surface samples, the top 50 taxonomic units that contributed to the difference were summarized and their abundance was plotted (Supplementary Figure S1). The result showed that the taxonomic units belonging to *Verrucomicrobia* and *Bacteroidetes* phylum were more abundant in WSP1. For example, the contribution of *Lentimonas* within the phylum *Verrucomicrobia* to the differences in each pairwise comparison between surface samples ranged from 0.742% to 1.325%, and that of *Polaribacter* and *Flavobacterium* (*Bacteroidetes*) ranged from 0.299% to 0.413% and 0.133% to 0.511% respectively. Furthermore, one group of Alphaproteobacteria (Rhodobacterales) was found to be more abundant in WSP2, WSP3 and WSP5 when compared with WSP1, and the contribution difference between WSP1 and other surface sites ranged from 0.331% to 0.452%. In contrast, the SARA11 clade in *Alphaproteobacteria* and *Oceanospirillales* in *Gamaproteobacteria* were more abundant in WSP2, WSP3 and WSP5.

### General Metabolism Categories

**FIGURE 5:**
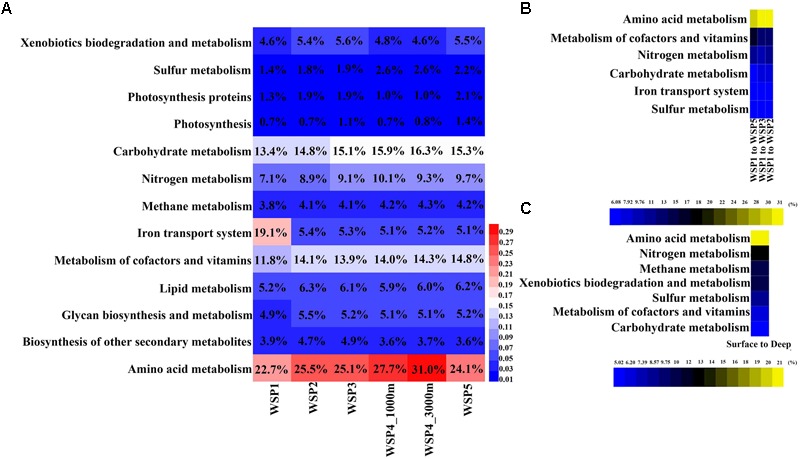
The relative abundance of functional genes in KEGG metabolism modules **(A)**, and their contribution to the difference between surface and deep waters **(B)**, and between the surface samples **(C)**. In each sample, the abundance of each KEGG module was calculated by summing the coverage of the genes in it, and then normalized by the sum of all functional genes’ coverage.

The annotated functional genes from each assembled contig were extracted and assigned to selected subsets of KEGG categories for subsequent distribution analysis of genes with general functions (**Figure 5A**). The coverage of each gene was calculated and summed when genes were assigned to the functional categories. It can be seen from the result that the amino acid metabolism was the most abundant functional category with relative abundance range from 14.33% to 18.31% within samples. The second most abundant functional category was carbohydrate metabolism at 13.24–16.11% followed by cofactor and vitamin metabolism at 7.32–11.32%. In order to identify the categories with significant contribution to the differences between the groups, the SIMPER analysis method was used. The functional categories that accounted for >5% variance were selected, and the value of their difference contribution was plotted (**Figures 5B,C**). The spatial comparison of functional categories between surface water samples (**Figure 5B**) showed that those involved in nitrogen metabolism, amino acid metabolism, the iron transport system, sulfur metabolism, and carbohydrate metabolism were significantly different between WSP1 and other surface samples. Using the average abundance of functional categories in the surface and deep groups, we found that the functional categories of amino acid metabolism, nitrogen metabolism, methane metabolism, xenobiotics biodegradation and metabolism, cofactors and vitamin metabolism, sulfur metabolism, and carbohydrate metabolism were significantly different between surface and deep groups (**Figure 5C**), with all these categories more abundant in the deep sea samples. Overall, three nutrient cycling categories (nitrogen metabolism, carbohydrate metabolism and sulfur metabolism), and the ‘metabolism of amino acids’ category exhibited significant differences when compared both vertically and spatially. The interconnection of the microbial taxa both within and between these various metabolism categories warrants further investigation.

### Nitrogen Cycling in the WSP

**FIGURE 6:**
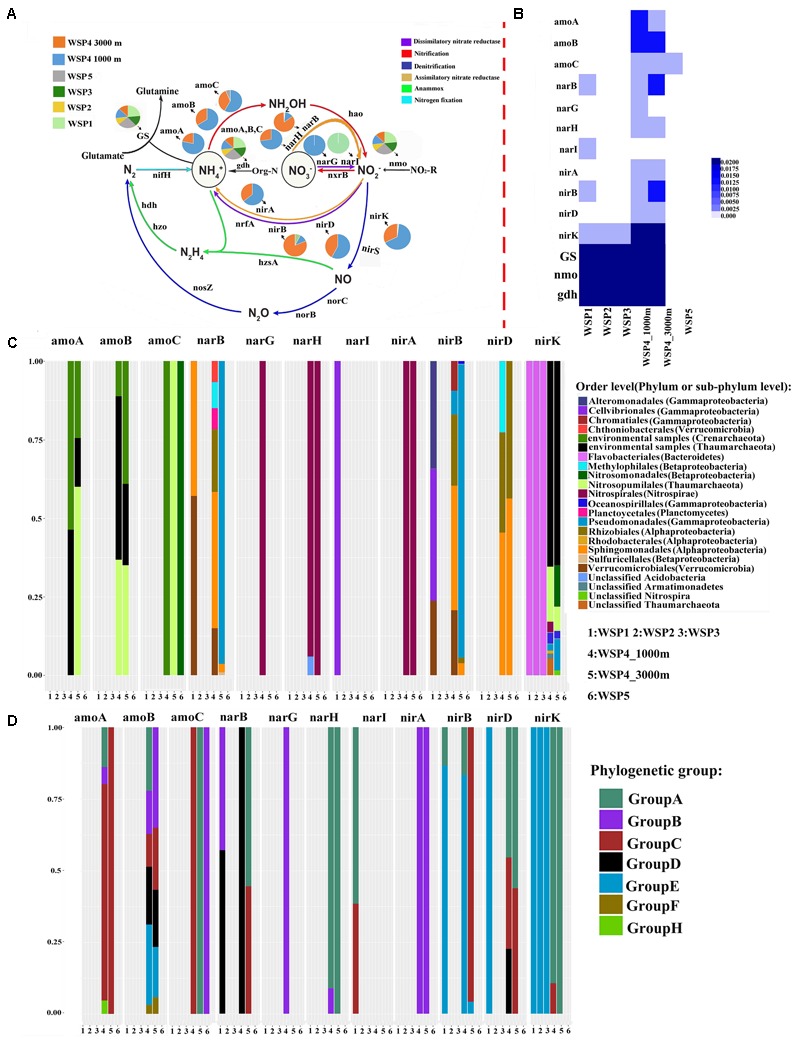
The pie charts represent the relative abundance of nitrogen metabolism related genes, which was calculated through dividing the sum of a gene’s coverage in an individual sample by the sum of this gene’s coverage in all samples **(A)**. The absolute abundance of each gene in each sample was calculated through dividing the coverage of an individual gene by the summed coverage of all functional genes, and the final value was squared **(B)**. The microbial taxa of the genes and its relative abundance in each sample **(C)**. The genotype of the key genes and its relative abundance in each sampling site **(D)**. The classification of each genotype was based on the phylogenetic tree of the reads derived from the metagenomic data and the reference sequences downloaded from NCBI. The phylogenetic tree was generated using the maximum likelihood method with 1,000-time bootstraps.

In total, four metabolic modes, including nitrification, denitrification, dissimilatory nitrate reductase, and assimilatory nitrate reductase are detected in our metagenomic dataset (**Figures 6A,B**). Three exogenous ammonia input-related genes, glutamine synthetase (*GS*), glutamate dehydrogenase (*gdh*) and nitronate monooxygenase *(nmo)* were widely distributed throughout the WSP (**Figure 6A**). Ammonia monooxygenase subunits A and B (*amoA* and *amoB*) were only detected in Group III, and only a small percentage of ammonia monooxygenase subunit C (*amoC*) was detected in WSP5. In addition, there was a clear trend that the relative abundance of all these ammonia monooxygenase subunits (**Figure 6A**) increased with depth. A heat map based on the relative abundance of each gene’s coverage, normalized by the summed coverage of all genes in each sample, shows that *gdh, GS* and *nmo* were more abundant than the other genes, and they were distributed in all the samples (**Figure 6B**). In general, the two normalization approaches showed a similar distribution pattern of the various nitrogen cycling genes.

To distinguish the genotypes and the affiliated microbial taxa of each nitrogen-related metabolism gene, the microbial taxa were extracted through MEGAN software, and the representative sequence from each clustered gene OTU was selected to construct the phylogenetic tree (Supplementary Figures S3–S13). The relative abundance of the microbial taxa in each gene was plotted (**Figure 6C**). Based on the constructed phylogenetic tree, each gene was then classified into several phylogenetic groups and the relative abundance of these groups was plotted (**Figure 6D**). The results showed that in WSP4 1000 m, the *amoA, amoB, nirD*, and *nirK* genotypes were more diverse than they were in WSP4 3000 m. The *amo* genes in deep water (Group III) were all detected in Archaea, whereas a small part of amoC gene was detected in WSP5, likely harbored by *Nitrosopumilales* in *Betaproteobacteria*. The microbial taxa of the *amoA* and *amoB* genes found in deep water were mainly *Crenarchaeote* and *Nitrosopumilales*. The most abundant phylogenetic group of *amoA* in deep water was groupC, water column bacteria III (WCB_III). The microbial taxa and phylogenetic type of *amoC* changed in different samples. For example, in WSP5 the *amoC* gene was mainly harbored by *Crenarchaeota* in group C, while in WSP4 3000m and WSP4 1000m it was mainly harbored by *Nitrosopumilales* in group A and *Nitrosomonadales* in group B, respectively. Furthermore, the microbial taxa and phylogenetic groups of *nirA* and *narH* were very similar in WSP4 1000 m and WSP4 3000 m; they were mainly harbored by *Nitrospirales* in the same phylogenetic type. Overall, the genes involved in the nitrification in the deep waters of the WSP were distributed into four distinct phyla (*Thaumarchaeote, Euryarchaeota, Gammaproteobacteria* and *Alphaproteobacteria*), whereas those involved in nitrate dissimilation/assimilation and denitrification in the WSP deep waters were mainly distributed into five distinct phyla (*Nitrospinae, Alphaproteobacteria, Gammaproteobacteria, Euryarchaeota* and *Thaumarchaeote*).

### Sulfur Metabolism-Related Functional Genes in the WSP

**FIGURE 7:**
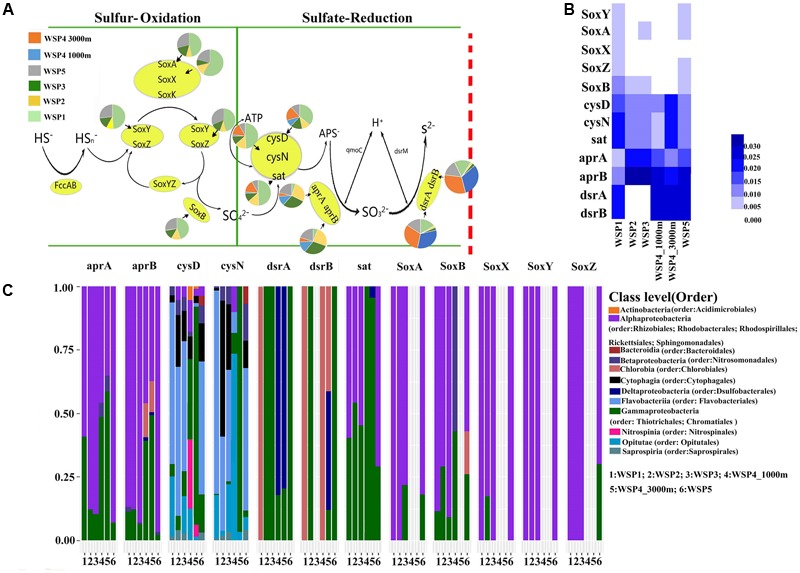
The pie charts represent the relative abundance of sulfur metabolism related genes, which was calculated through dividing the sum of a gene’s coverage in an individual sample by the sum of this gene’s coverage in all samples **(A)**. The absolute abundance of each gene in each sample was calculated through dividing the coverage of an individual gene by the summed coverage of all functional genes, and the final value was squared **(B)**. The microbial taxa of the genes and its relative abundance in each sample **(C)**. The taxonomic identifications were based on the blast results of NR database.

The sulfur cycle is predominated by two microbial metabolic processes, sulfur oxidation and sulfate reduction (Cao et al., 2014). Our results (**Figures 7A,B**) showed that the sulfur oxidation genes were mainly detected in the surface samples (WSP1, WSP2, WSP3, WSP5), whereas the sulfate reduction genes were more abundant in all samples except the WSP1 sampling station. The sulfur oxidation genes, including *SoxXYZAB*, were only detected in the surface water and found to be mainly harbored by *Alphaproteobacteria* and *Gammaproteobacteria*. The *cysD* and *cysN* genes had a very diverse and similar microbial taxa distribution between WSP1, WSP2, WSP3 and WSP5. In addition, the *dsrA* and *dsrB* gene were all harbored by *chlorobia* in WSP1, while in other surface samples the genes were mainly harbored by *Gammaproteobacteria* (**Figure 7C**).

### CAZymes and Iron Metabolism

**FIGURE 8:**
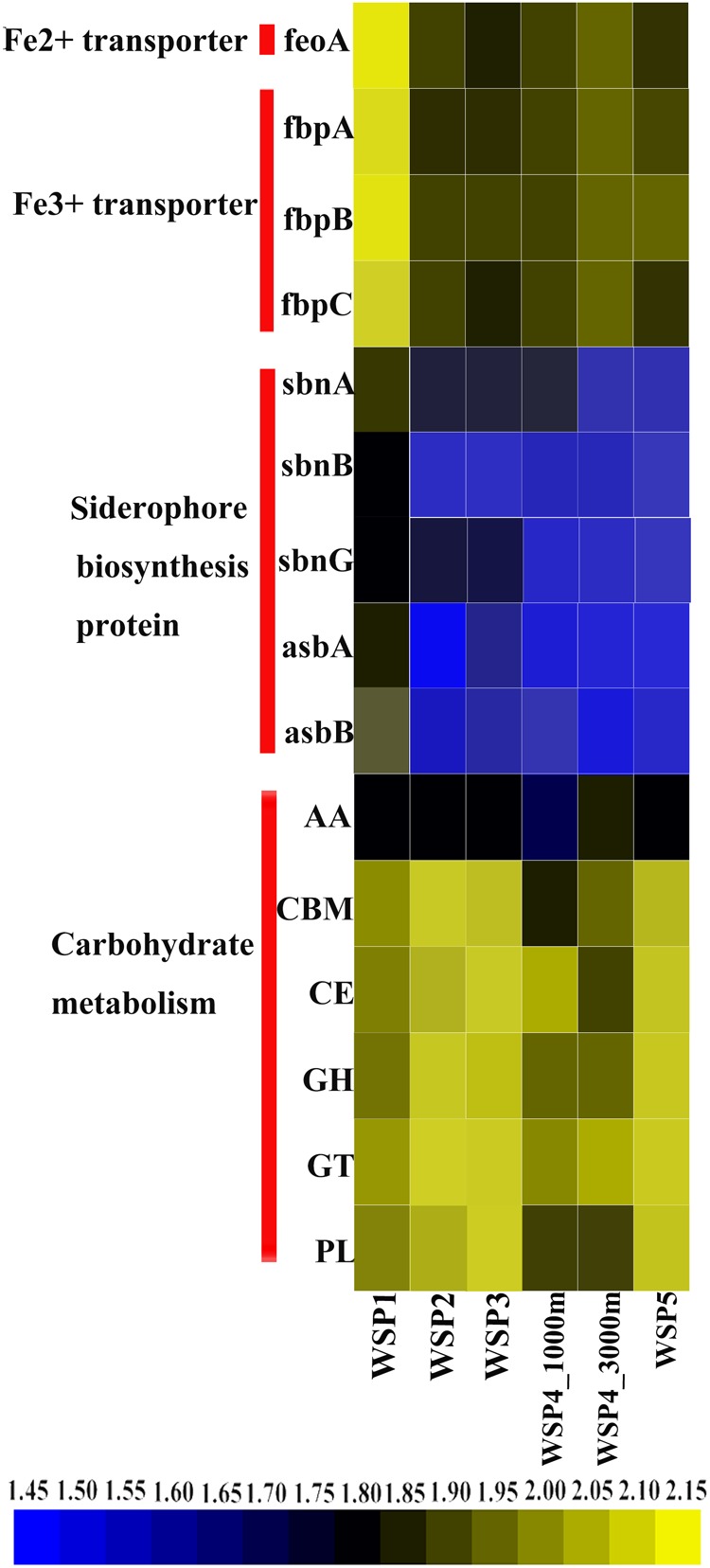
The relative abundance of carbohydrate metabolism modules and iron metabolism related genes in each sample. Abundances were based on the calculation of the genes’ coverage, and the abundance value was first log scaled and then squared.

To understand the main carbohydrate metabolism process in each sampling site, the predicted ORF in each sample was annotated via the CAZymes database (**Figure 8**), as follows: GHs (glycoside hydrolases); GTs (glycosyltransferases); AAs (auxiliary activities); CEs (carbohydrate esterases); CBMs (carbohydrate binding modules); and PLs (polysaccharide lyases) (Lombard et al., 2014). After the coverage of each gene in carbohydrate metabolism categories was summed, the abundance of each category was plotted (**Figure 8**). The result showed that carbohydrate metabolism encoding genes were more abundant in WSP2, WSP3, and WSP5 when compared with WSP1. Based on the annotation of the NR database, the abundance of siderophore biosynthesis protein, Fe^2+^ transporter and Fe^3+^ transporter-encoding genes was also investigated and plotted (**Figure 8**). The results indicate that all these iron metabolism related genes were very abundant in WSP1 compared with other sampling sites.

### Correlations Between the Taxonomic Taxa

**FIGURE 9:**
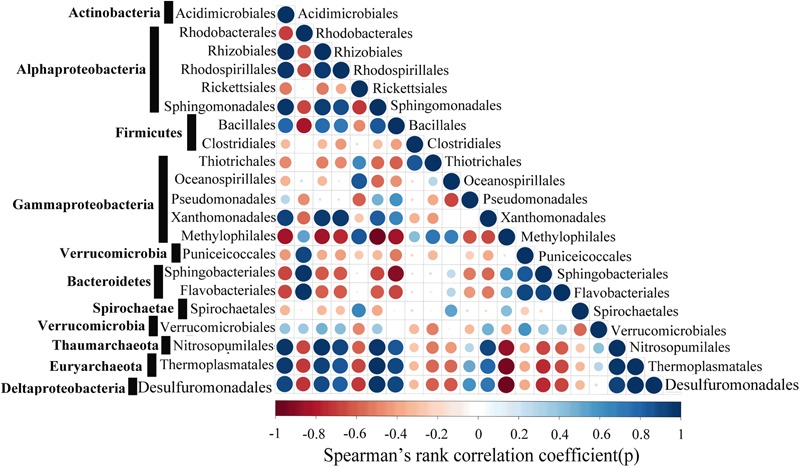
Network co-occurrence patterns with Spearman’s *P-*value < 0.01. Each dot represents a significant correlation between two microbial taxa with red and blue representing negative and positive correlations, respectively.

Strong positive correlations between the *Acidimicrobiales, Rhizobiales, Rhodospirillales, Sphingomonadales, Bacillales, Xanthomonadales, Nitrosopumilales, Thermoplasmatales* and *Desulfuromonadales* were detected by correlation analysis (**Figure 9**). The Spearman’s rank correlation coefficients (SRCC) of these 9 orders were all >0.4. In addition, a stronger correlation was observed between *Acidimicrobiales, Nitrosopumilales, Thermoplasmatales* and *Desulfuromonadales*, with SRCC values between them >0.65, and these 4 orders are known to actively participate in the metabolism of nitrogen, sulfur, and carbohydrate. It is also worthy to note that six orders (*Rhodobacterales, Rickettsiales, Thiotrichales, Methylophilales, Sphingobacteriales, Flavobacteriales*) were significantly negatively correlated with the selected microbial taxa of *Thaumarchaeote, Euryarchaeota* and *Deltaproteobacteria*, with SRCC values between them <-0.4.

## Discussion

Advances in sequencing technologies and data analysis approaches have made investigations into the diversity of microbes and their associated versatile metabolisms more straightforward and accurate. For example, the application of advanced sequencing technology has produced substantial amounts of information regarding the diversity and metabolism of microbes in the eastern subarctic Pacific Ocean (Cochlan, 2001; Van Mooy et al., 2004). In contrast, the taxonomic diversity and the metabolism potential of the microbial community in the WSP Ocean have been scarcely reported until now. Therefore, in this study, during a research cruise of the WSP in June 2014, five stations were selected for spatial and vertical sampling. Using a metagenomics approach, we obtained a total of 681,578 contigs (>500 bp) and 10,850,291 protein-encoding genes. The data presented here provide a substantial amount of functional and taxonomic information of microorganisms across the geographic boundary and vertical waters in the WSP.

### Nutrient Metabolism in the WSP

#### Amino Acid Metabolism

While amino acids can be used as a source of energy for microorganisms (Stocker et al., 2008), they can also be utilized directly to synthesize proteins. Therefore, the metabolism of amino acids might vary significantly in diverse environments (Gianoulis et al., 2009). It has been reported that the energy cost of finding metals for use in cofactors might be higher than synthesizing the transport machinery and degradative components required for importing exogenous amino acids. In the latter situation, the requirement for cofactors is reduced. Thus, importing extracellular amino acids might be more favorable than direct protein synthesis, especially in oligotrophic environments where the source of material for producing the cofactors required is somewhat limiting (Gianoulis et al., 2009). Consistent with previous reports, in our study the sequences assigned to amino acid metabolism (especially for amino acid degradation), were shown to be significantly enriched in WSP1, whereas the biosynthesis-related categories for amino acid metabolism were more abundant in the WSP2, WSP3, WSP5 (Supplementary Figure S14). This might be an energy-preserving mechanism used by microorganisms in the relatively oligotrophic conditions (WSP1).

#### Nitrogen Metabolism

With regards to the metabolism of nitrogen, it is clear that nitrogen cycling in the WSP is mainly conducted in the deep sea because the nitrogen cycling genes were in general detected in deep waters (**Figure 6**). In a previous report, the *Nitrosopumilus maritimus* SCM1 isolate was demonstrated to harbor all the putative ammonia monooxygenase genes (i.e., *amoA, amoB* and *amoC*), which were organized in one operon (Walker et al., 2010). Similarly, *Nitrosopumilales* in WSP4_3000 m was detected to have a complete putative ammonia monooxygenase operon in one assembled contig, and the genome of this Archaea had been extracted from the metagenomic data (data not published). According to our analysis, the ammonia monooxygenase-encoding genes detected in the deep waters were all from Archaea. These results are consistent with a previous report, which indicated that Archaea plays an important role in ammonia oxidation in the open ocean (Mincer et al., 2007). The genotypes of *amoA* in deep waters are mainly assigned into the WCB_I and WCB_III groups, which is consistent with a former classification indicating that these two groups are mainly distributed in the eastern tropical North Pacific Ocean (ETNP) (Francis et al., 2005) and the Arctic (Pedneault et al., 2014). In addition, in previous studies the *Thaumarchaeota* was considered to be one class branch of the *Crenarchaeota*, and *Crenarchaeota* reference sequences in the phylogenetic tree of nitrogen genes all follow this classification system (Brochier-Armanet et al., 2008; Spang et al., 2010). Therefore, the *amoA* sequences in our data should belong to *Thaumarchaeota* rather than *Crenarchaeota* (Supplementary Figure S2). The abundant *nmo* (nitronate monooxygenase) and *gdh* (glutamate dehydrogenase) genes in the surface samples indicate that the metabolism of organic nitrogen was prevalent in the surface waters. In addition, the abundant *GS* (glutamine synthetase), and less abundant ammonium oxidation genes in the surface waters, reflects the light inhibition of ammonium oxidation in the surface layer. Light is thought to inhibit both ammonia-oxidizing Archaea (AOA) and bacteria (AOB) (Horrigan and Springer, 1990; Guerrero and Jones, 1996; Church et al., 2010; French et al., 2012; Merbt et al., 2012), but nitrification can be detected in the euphotic zone (Wankel et al., 2007; Santoro et al., 2010), which could be attributed to different light sensitivities of AOA and AOB resulting from a potential mechanistic difference between their respective versions of AMO (French et al., 2012; Merbt et al., 2012).

#### Sulfur Metabolism

It has been previously reported that eight moles of electrons are generated during the complete oxidation of one mole of thiosulfate or sulfide to sulfate. This is a common mechanism used by chemolithoautotrophs for energy production (Shao et al., 2010). In our study, we detected a truncated sulfur oxidation (Sox) system in the surface water of the WSP. This is similar to other recent studies that reported the Sox system in a coastal area (Murillo et al., 2014). It is reported that due to a lack of the sulfur dehydrogenase complex, the direct oxidation of sulfone sulfur atoms to sulfate is prevented; consequently, the truncated form will only produce two moles of electrons (Ghosh and Dam, 2009). However, although this incomplete form of Sox does not allow lithotrophic microorganisms to utilize thiosulfate as their sole source of energy, the oxidizing thiosulfate can still be processed at low rates (Friedrich et al., 2001). Furthermore, *Alphaproteobacteria* might play an irreplaceable role in sulfur metabolism (especially in WSP2, WSP3, and WSP5), as it harbors sufficient genes for both sulfur oxidation and sulfate reduction, including genes encoding dissimilatory sulfite reductase (*dsrA* and *dsrB*). This finding is consistent with a previous report, which indicates that sulfur-oxidizing *Alphaproteobacteria* with a chemolithoheterotrophic lifestyle might be abundant in the non-hydrothermal sediment and water column (Meyer and Kuever, 2007).

#### Carbohydrate and Iron Metabolism

Our results indicate that the relative high abundance of carbohydrate metabolism-encoding genes in the surface layer may reflect that the organic matter in the surface water of the WSP is widely utilized by the resident microorganisms (Li et al., 2016). Compared to WSP1, the higher relative abundance of these genes in WSP2, WSP3, and WSP5 may be caused by elevated levels of phytoplankton-derived particles (Unanue et al., 1998). Siderophores are organic compounds which will be produced by microorganisms and plants under low-iron conditions. The main function of these compounds is to chelate the ferric iron [Fe(III)] from environments and thereby make it available for microbial and plant cells (Miethke and Marahiel, 2007). The relative high abundance of Fe^2+^, Fe^3+^ and siderophore biosynthesis protein-encoding genes in WSP1 indicates the relatively iron-limited conditions of this site’s surface water (Orlova et al., 2004; Suzuki et al., 2011). The strong mixing of iron-rich water from the Sea of Okhotsk at the Bussol Strait relieves the severity of iron limitation, which causes a low abundance of these genes in other surface samples.

### Taxonomic and Functional Variation in Water Column

In the WSP, the microbial communities at the surface were markedly different from those in deep waters. For instance, we showed that Archaea are significantly enriched in the deep waters, whereas bacteria are more abundant at the surface (**Figure 4**). These results support a previous report indicating that the abundance of Archaea increases with ocean depth (Hewson et al., 2009). Additionally, the deep-sea abundant Archaea, *Thaumarchaeota*, showed a strong positive correlation with *Sphigomonadales* (**Figure 9**). *Thaumarchaeota* marine group I was widely known to be an ammonia oxidizer in the marine environment. Indeed, consistent with former metagenomic study of the deep water of North Pacific Subtropical Gyre (Konstantinidis et al., 2009), the *amo* genes (*amoA, amoB*, and *amoC*) (Supplementary Figures S3–S5) were more abundant in deep water (1000 and 3000 m). However, in our samples it was detected mainly in *Nitrosopumilales* (a member of *Thaumarchaeota* marine group I), while in the North Pacific Subtropical Gyre it was mainly detected in the ammonia-oxidizing bacteria like *Beta*- and *Deltaproteobacteria*. On the other hand, the dissimilatory nitrate reductase (*narB*) and nitrite reductase (*nirB* and *nirD*) encoding genes were widely found in the *Sphingomonadales* and *Pseudomonadales* bacteria, both in this study and in previous reports (Bala et al., 2010), which suggest that the products of bacteria ammonia oxidation might undergo further processing by *Sphingomonadales* and *Pseudomonadales* in the deep waters. The co-occurrence of *Thaumarchaeaota, Sphingomonadales*, and *Pseudomonadales* in the deep waters suggests that they were closely associated and perform different roles in nitrogen cycling. Similarly, a recent study also reported a nitrogen metabolism related nutrient-source exchange between *Candidatus Dadabacteria, Deltaproteobacteria* and *Thaumarchaeota* in the sediment from an alluvial aquifer (Hug et al., 2016). *Candidatus Dadabacteria* and *Deltaproteobacteria* harbor the dissimilarity nitrate reduction to ammonia (DNRA) pathways, whereas *Thaumarchaeota* harbors a complete ammonia monooxygenase (*amoA,B,C*) operon.

Results showed that in the surface water, both *Flavobacteriales* and *Synechococcaceae* had higher relative abundance (Supplementary Figure S2). The high abundance of the former might be due to the fact that this bacteria prefers to utilize complex organic matter by attaching directly to algal cells as well as to algal-derived detrital particles (Kirchman, 2002; Teeling et al., 2012). In previous studies, *Flavobacteriales* was reported to harbor both sulfur-oxidizing and sulfate-reducing genes (Wu et al., 2015; Shimizu et al., 2016), and it showed a relatively low abundance across the northeastern Pacific Ocean, accounting less than 5% of the community (DeLong et al., 2006; Biers et al., 2009). In our study, abundant sulfur metabolism genes were mainly detected in *Flavobacteriales*, and it accounted for 8.4–14.2% of all microbes in the surface water of the WSP (Supplementary Figure S2). Thus, the surface abundant *Flavobacteriales* might not just utilize complex organic matter derived from phytoplankton, it might also play a role in sulfur oxidation and sulfate reduction. Furthermore, *Prochlorococcus* was reported to be the most abundant and active cyanobacteria in the North Pacific Subtropical Gyre (DeLong et al., 2006; Frias-Lopez et al., 2008). However, in our result the *Synechococcaceae* was found to be more abundant in the surface water of the WSP, which may be because *Prochlorococcus* is more abundant within the 40°S to 40°N latitudinal band of oceans and nutrient-deprived environments (Partensky et al., 1999). In addition, the *Actinobacteria* could account for nearly 6–15% across the subtropical Pacific Ocean (Biers et al., 2009), whereas in the WSP the abundance of *Actinobacteria* was found to account for less than 2% of the community in the surface and 1–4% in the deep water.

### Taxonomic and Functional Variation Associated With Current Transformation

The surface water microbial communities also displayed spatial variation in their composition in the WSP. Our finding that SAR11 is very abundant in the WSP, especially WSP1, is very similar to the Northeastern Pacific Ocean (Biers et al., 2009), and also agrees with the previous reports that SAR11 is widely distributed around the global ocean and more prevalent in an oligotrophic environment (Giovannoni et al., 2005; Bianchi, 2011). Comparing with WSP1, samples from the Sea of Okhotsk (WSP5) and Oyashio current (WSP2 and WSP3) were found to have a higher abundance of *Rhodobacterales* (Supplementary Figure S2), which took up 4.2–5.8% of the microbial community, even higher than the reported average in coastal waters (3.8%) (DeLong et al., 2006). This may reflect the nutrient-rich environmental conditions of the Sea of Okhotsk and Oyashio current, and is also consistent with the previous report that *Rhodobacterales* prefers a nutrient-rich environment (Azam and Malfatti, 2007). Since the *dsrA* and *dsrB* geneharboring bacteria could use intermediate valence sulfur species as terminal electron acceptors of its sulfate-reducing process, the low abundance of these two genes in WSP2 and WSP3, where strong water mixing occurs, could be explained by a more strong oxidation of the intermediate valence elements, such as S (-II) and Fe (II) (Bagnoud et al., 2016). Without strong mixing in other sampling sites, the abundance of *dsrA* and *dsrB* gene is relatively higher.

The finding of the dramatic change in the abundance of amino acid and carbohydrate metabolism categories between WSP1 and other surface samples varies greatly from the result of a previous study conducted in the Central Pacific Ocean, where genes of these two metabolisms have no discernible differences among their samples (Hewson et al., 2009). This difference could be caused by the nutrient and iron-rich water in the Sea of Okhotsk and Oyashio current, which first promotes the growth of phytoplankton, subsequently increases the abundance of phytoplankton-derived particles, and finally causes the change in gene abundance of microbial carbohydrate metabolism categories. In a previous report, the phytoplankton derived particles could increase amino acid uptake rate of bacteria (Unanue et al., 1998), which is also in agreement with the low abundance of amino acid degradation and transporter encoding genes in WSP2, WSP3, and WSP5.

## Conclusion

In summary, microbial communities in seawater at four surface sites and two deep-sea samples in the WSP were studied using a metagenomics approach, for the first time, to elucidate the taxonomic and functional diversity in this highly dynamic region. The result showed that Archaea is more abundant and enriched in the deep waters. When compared to the ammonia-oxidizing microorganisms in the North Pacific Subtropical Gyre, the ammonia monooxygenase was mainly harbored by Archaea (*Thaumarchaeota*) in the WSP instead of bacteria. The products of ammonia oxidation from *Thaumarchaeota* could be further processed by *Sphingomonadales* and *Pseudomonadales* in the WSP, evidenced not only by their positive relationship with *Thaumarchaeota*, but also for carrying dissimilatory nitrate reductase (*narB*) and nitrite reductase (*nirB* and *nirD*). In addition, unlike the subtropical Pacific Ocean and northeastern Pacific Ocean, the WSP harbors more *Flavobacteriales*, and they may play an important role in utilizing the organic matter derived from phytoplankton, sulfur oxidation, and sulfate reduction. Our results imply that the exchange of water from the Sea of Okhotsk may not only transform the East Kamchatka Current to the Oyashio Current, but also significantly change the composition and functional genes of the microbial community in the WSP. Furthermore, the wide distributions of nutrient cycling genes in diverse microbial taxa and the strong correlations between these taxa indicate that the nutrient metabolism processes of these microbial taxa in the WSP might be tightly coupled. Together, this study extended our knowledge of the microbial community structure and its associated metabolisms in the WSP, with a highlight on their differences in different ocean currents and depths. These findings provide evidence of how productive marginal seas could influence the microbial community composition and function, as well as biogeochemical cycling in the oceanic basin.

## Author Contributions

HL conceived and designed the experiments. KS and XX performed the experiments. YL analyzed the data. HL and HJ contributed reagents, materials and analysis tools. YL wrote the paper. HL, XX, and HJ contributed writing and analysis guidance. SC contributed database.

## Conflict of Interest Statement

The authors declare that the research was conducted in the absence of any commercial or financial relationships that could be construed as a potential conflict of interest.

## Footnotes

1 www.arb-silva.de
