# Supplementary material for: Metagenomic Insights Into the Microbial Community and Nutrient Cycling in the Western Subarctic Pacific Ocean
Source: Front Microbiol. 2018 Apr 4;9:623. doi: 10.3389/fmicb.2018.00623 (PMC5894113; doi:10.3389/fmicb.2018.00623)

**Table S1** The summary of sampling sites and nutrient level

| **Station** | **Date**  **(d/m/y)** | **Coordinates** | **Sample Depth (m)** | **T (ºC)** | **Salinity**  **(psu)** | **N+ N**  **(umol/kg)** | **NO_2_**  **(umol/kg)** | **NH_4_**  **(umol/kg)** | **PO_4_^-^**  **(umol/kg)** | **Si**  **(umol/kg)** |
| --- | --- | --- | --- | --- | --- | --- | --- | --- | --- | --- |
| WSP1 | 12/06/2014 | 53° 59'28.20"N,  162° 22'30.60"E | 1.5 | 1.811 | 32.365 | 0.1 | <0.01 | <0.2 | 0.13 | <0.5 |
| WSP2 | 11/06/2014 | 46° 36'36.06"N,  152° 3'7.20"E | 1.7 | 3.409 | 32.784 | 14.6 | 0.14 | <0.2 | 0.99 | 26.4 |
| WSP3 | 28/06/2014 | 45° 25'N,  153° 0'E | 1.8 | 7.282 | 32.828 | 13.07 | 0.18 | <0.2 | 1.21 | 10.8 |
| WSP5 | 2/07/2014 | 45° 43'16.14"N, 147° 56'36.78"E | 1.5 | 9.034 | 32.133 | NA | NA | NA | NA | NA |
| WSP4 1000_m | 29/06/2014 | 45° 0'N,  153° 30'E | 1000 | 3.280 | 32.811 | 43.61 | 0.02 | <0.2 | 3.23 | 95.5 |
| WSP4 3000_m | 29/06/2014 | 45° 0'N,  153° 30'E | 3000 | 3.335 | 32.886 | 42.65 | 0.02 | <0.2 | 3.10 | 97.3 |

**Table S2** Summary of sequence assembly and gene prediction.

| **Contig information** | **WSP1** | **WSP2** | **WSP3** | **WSP5** | **WSP4_1000 m** | **WSP4_3000m** |
| --- | --- | --- | --- | --- | --- | --- |
| **Total contigs (≥500bp)** | 108,849  (159.7Mb) | 133,261  (228.7Mb) | 115,970  (201.3Mb) | 136,635 (249.8Mb) | 108,849  (159.7Mb) | 78,014  (107.1Mb) |
| **2.5 kbp>** | 10,769 | 18,042 | 14,639 | 18,342 | 10,768 | 7,169 |
| **Size ≥ 25 kbp** | 163 | 300 | 421 | 630 | 163 | 36 |
| **Average size of contig(bp)** | 1,466 | 1,716 | 1,736 | 1,828 | 1,466 | 1,373 |
| **Largest contig (kb)** | 82 | 140.3 | 272.9 | 236.1 | 81.9 | 391.9 |
| **GC content (%)** | 43.78 | 40.1 | 37.56 | 41.32 | 43.78 | 43.92 |
| **Average sequence coverage** | 23.8 | 15.9 | 18.9 | 16.9 | 16.6 | 13 |
| **Highest sequence coverage** | 839 | 808 | 902 | 790 | 792 | 5173 |
| **Protein-coding genes** | 1,821,684 | 2,186,342 | 1,859,319 | 2,384,840 | 1,530,596 | 1,067,510 |
| **Genes in COGs** | 518,658 | 537,585 | 518,320 | 587,019 | 623,371 | 544,060 |
| **Total functional genes annotated in kegg** | 1,391,850 | 1,463,613 | 1,389,496 | 1,508,975 | 1,053,837 | 752,306 |

**Table S3** Kaiser-Meyer-Olkin and Bartlett’s test on two groups cluster in hierarchical cluster analysis

| **Kaiser-Meyer-Olkin Measure of Sapling Adequacy** | | .891 |
| --- | --- | --- |
| **Bartlett’s test of Sphericity** | Approx. Chi-Square | 521258.559 |
|  | df | 21 |
|  | Sig | .000 |

**Table S4** Rotated component matrix of two groups cluster in hierarchical cluster analysis

|  | **Component** | |
| --- | --- | --- |
|  | **1** | **2** |
| **WSP1** | .814 |  |
| **WSP2** | .806 |  |
| **WSP3** | .822 |  |
| **WSP5** | .801 |  |
| **WSP4_1000m** |  | .842 |
| **WSP4_3000m** |  | .946 |

**Table S5** Kaiser-Meyer-Olkin and Bartlett’s test on two surface groups cluster in hierarchical cluster analysis

| **Kaiser-Meyer-Olkin Measure of Sapling Adequacy** | | .841 |
| --- | --- | --- |
| **Bartlett’s test of Sphericity** | Approx. Chi-Square | 2046.062 |
|  | df | 6 |
|  | Sig | .000 |

**Table S6** Rotated component matrix of two surface groups cluster in hierarchical cluster analysis

|  | **Component** | |
| --- | --- | --- |
|  | **1** | **2** |
| **WSP1** |  | .977 |
| **WSP2** | .971 |  |
| **WSP3** | .853 |  |
| **WSP5** | .946 |  |

**Fig. S1** The microbial taxa that most contributed to the difference among surface samples

**
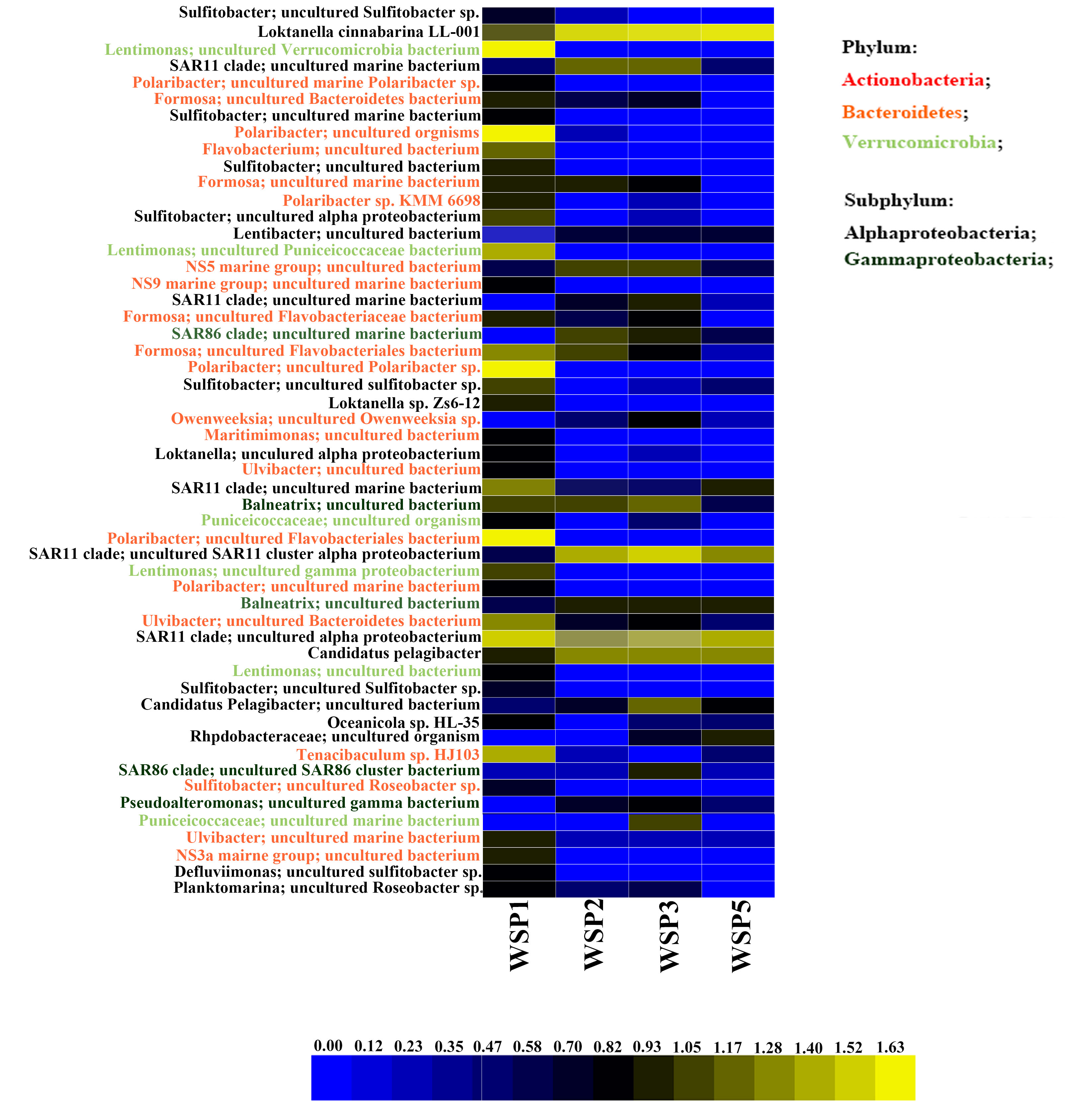
**

**Fig. S2** The order level community structure of protokaryon

**
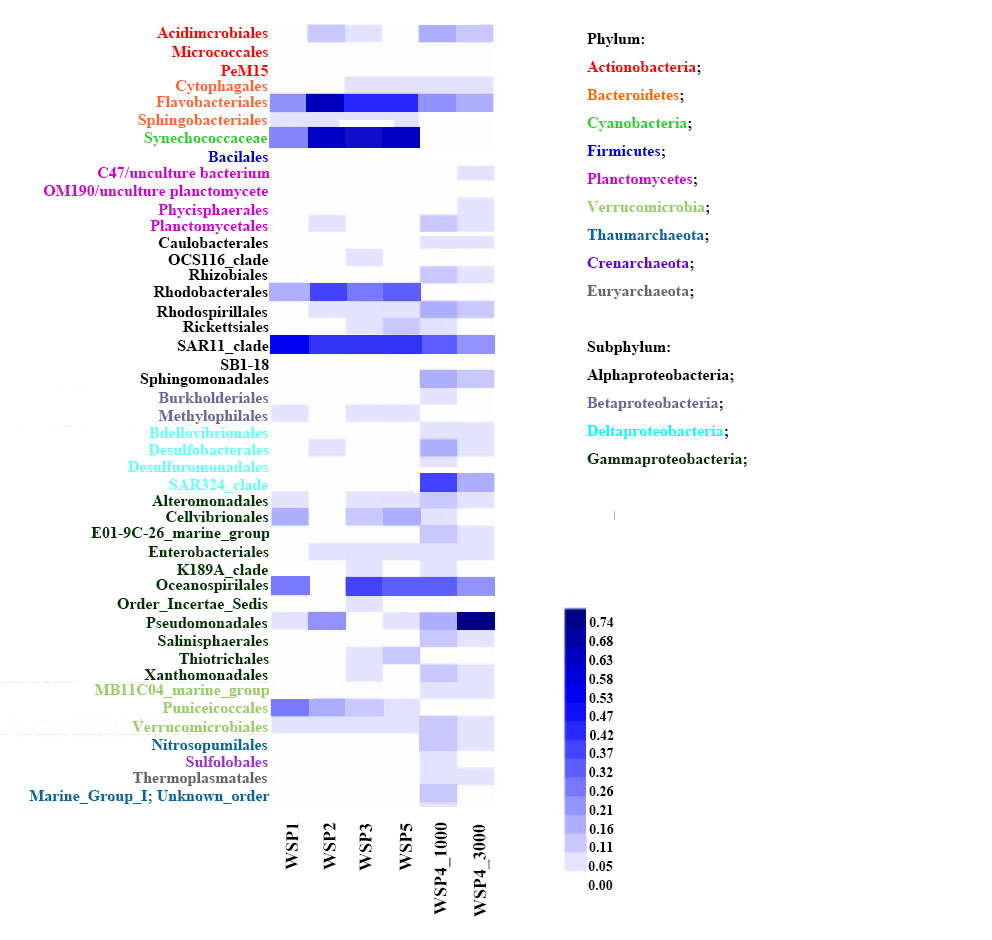
**

**Fig. S3** Phylogenetic tree to show the clustering of the *amoA* gene


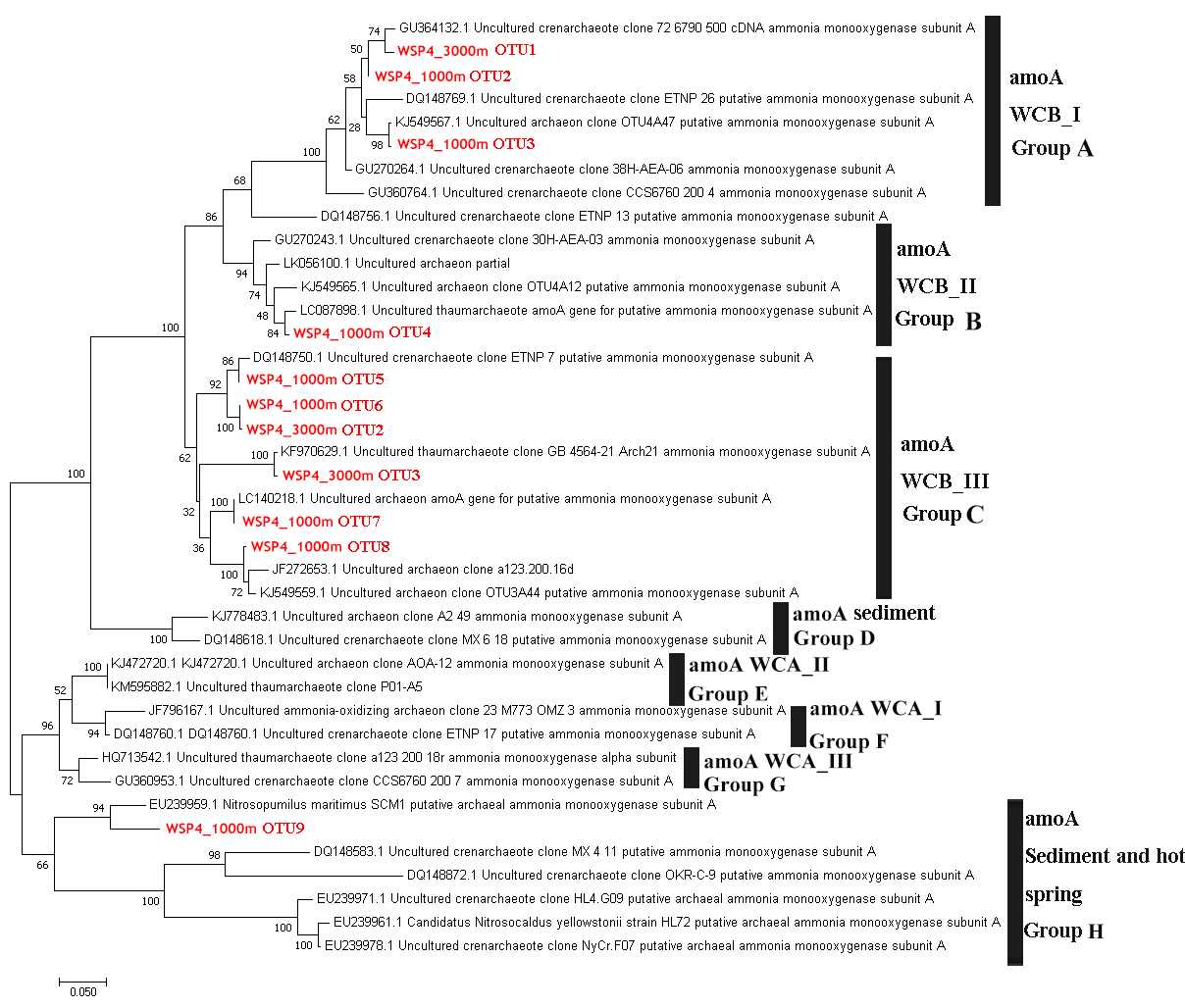


**Fig. S4** Phylogenetic tree to show the clustering of the *amoB* gene


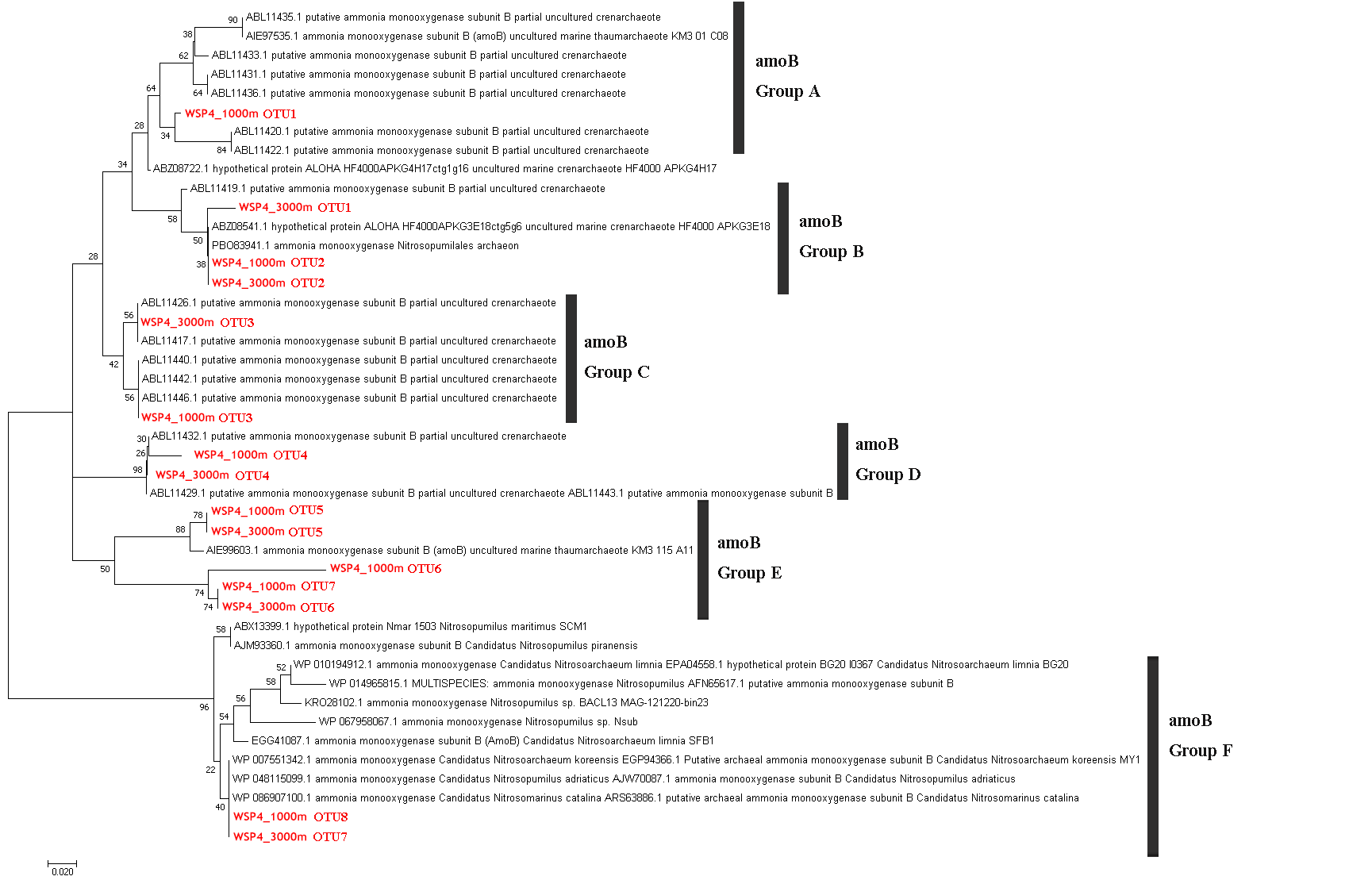


**Fig. S5** Phylogenetic tree to show the clustering of the *amoC* gene


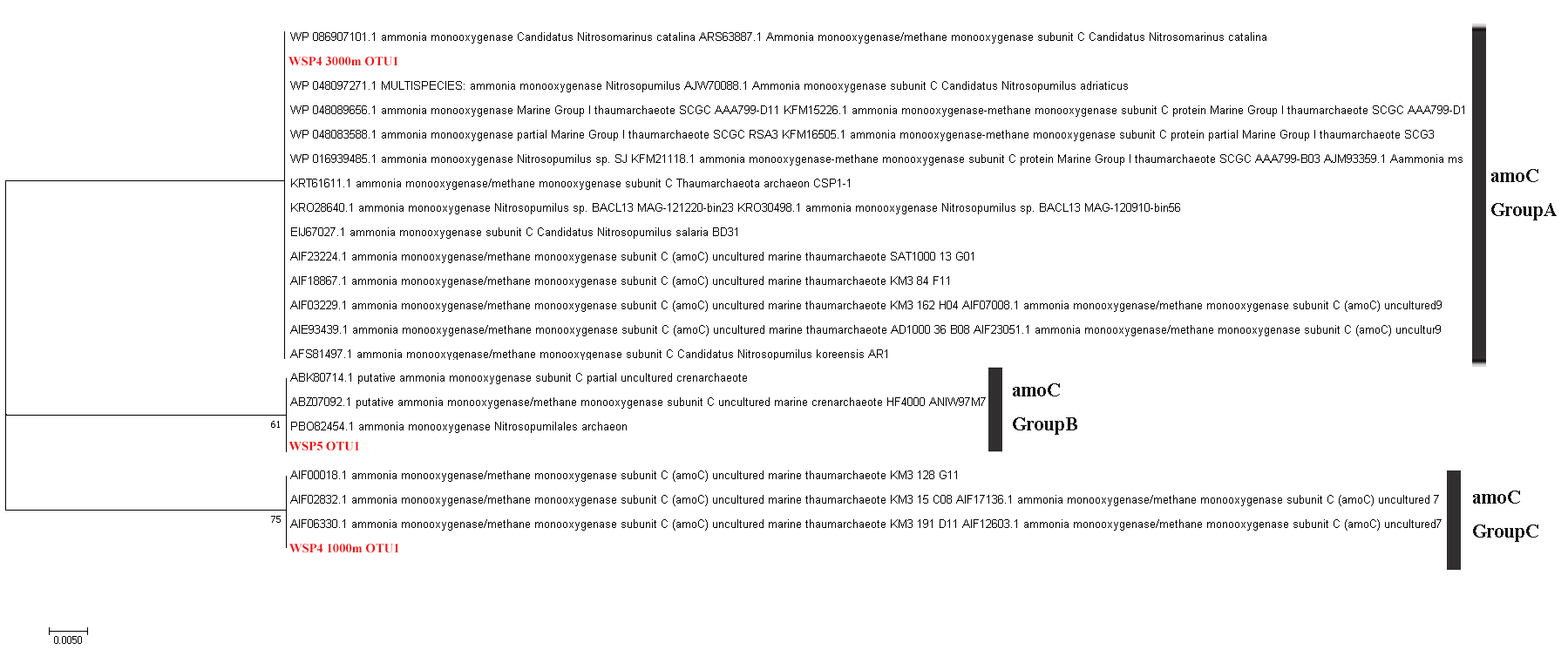


**Fig. S6** Phylogenetic tree to show the clustering of the *narB* gene


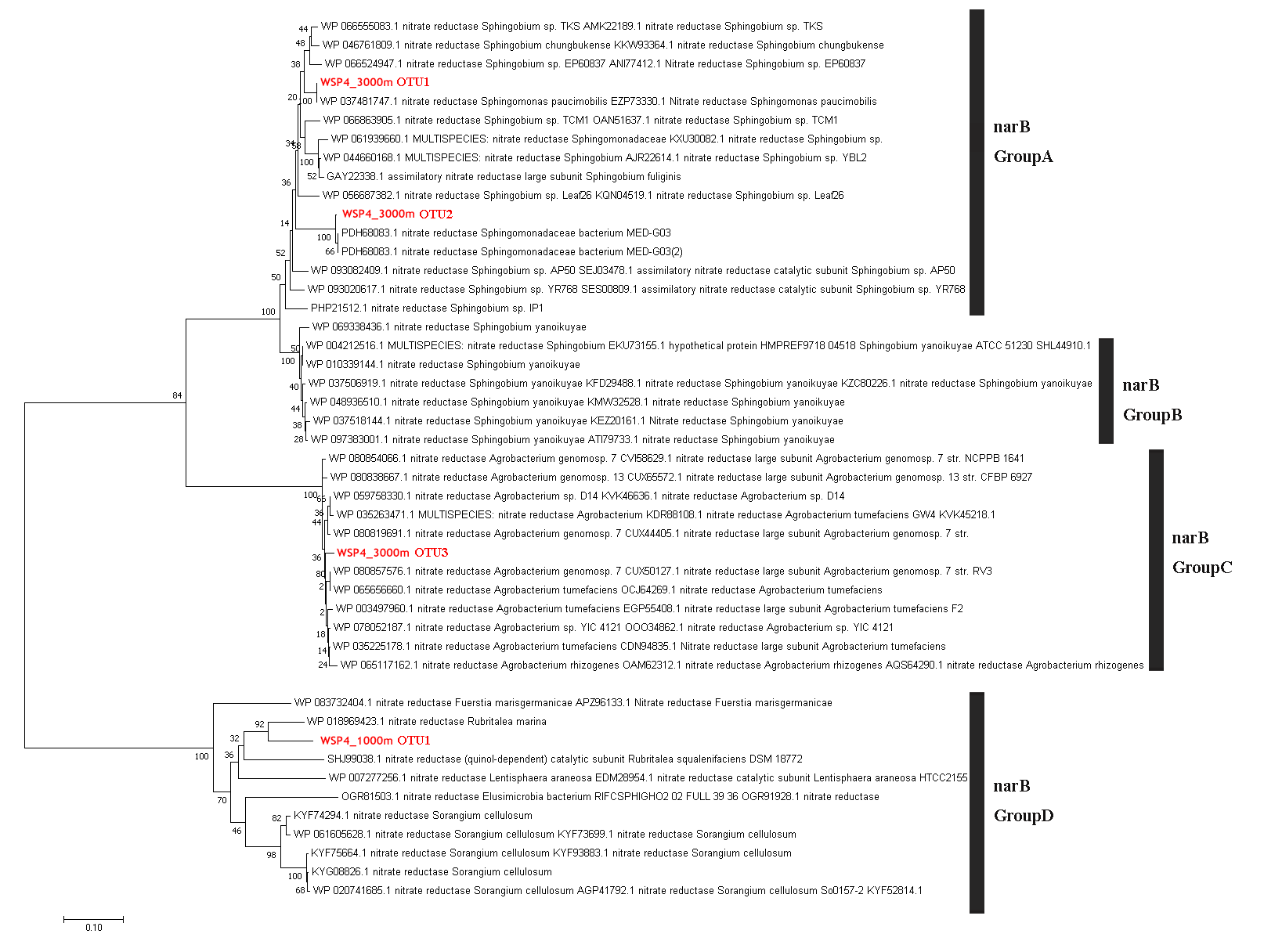


**Fig. S7** Phylogenetic tree to show the clustering of the *narG* gene


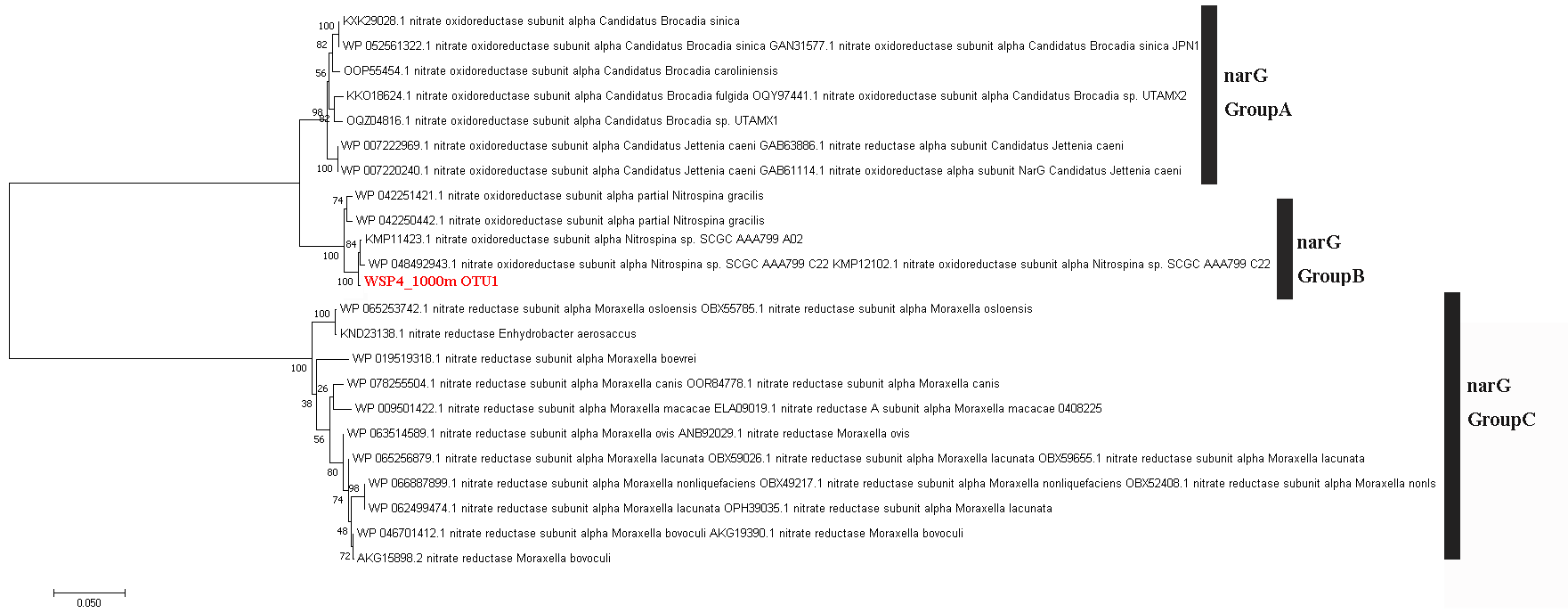


**Fig. S8** Phylogenetic tree to show the clustering of the *narH* gene

**
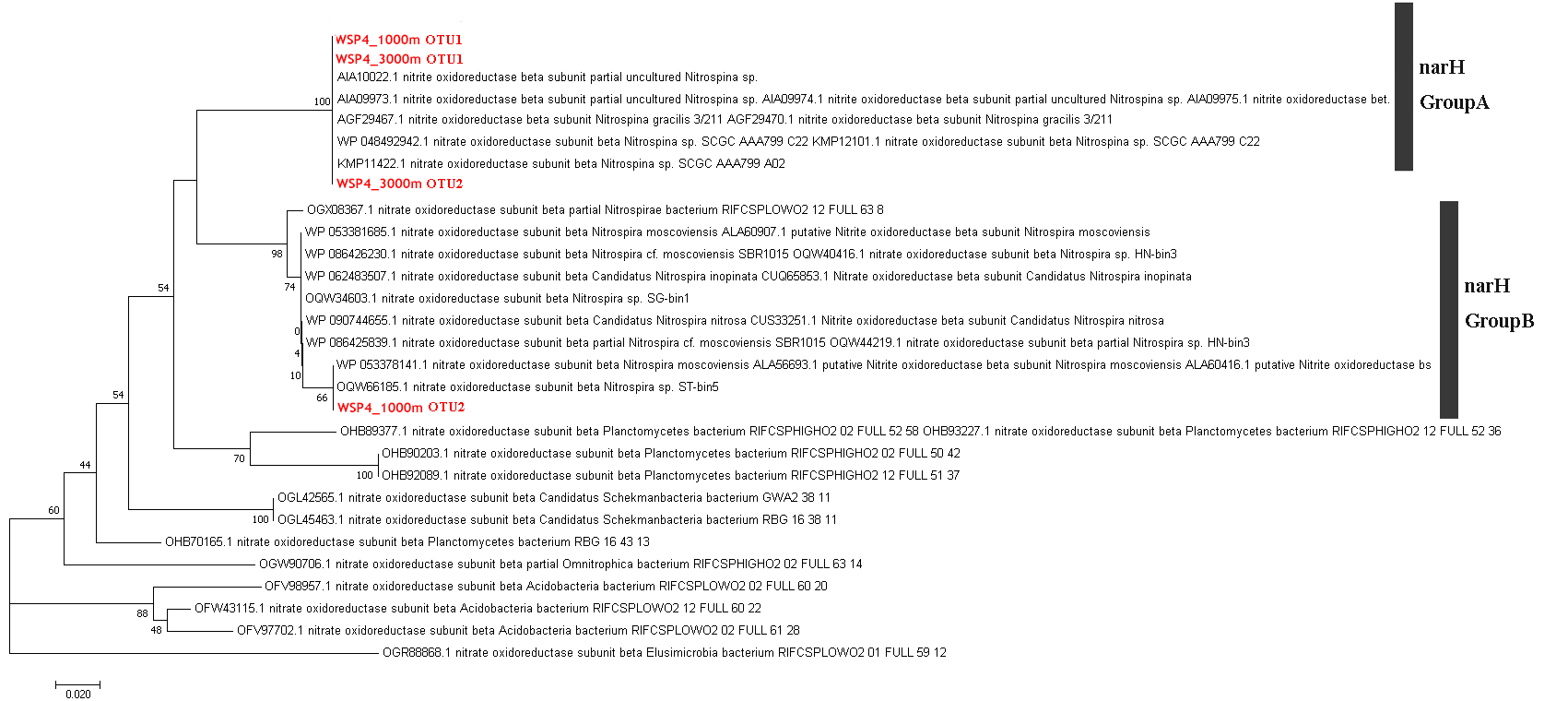
**

**Fig. S9** Phylogenetic tree to show the clustering of the *narI* gene

**
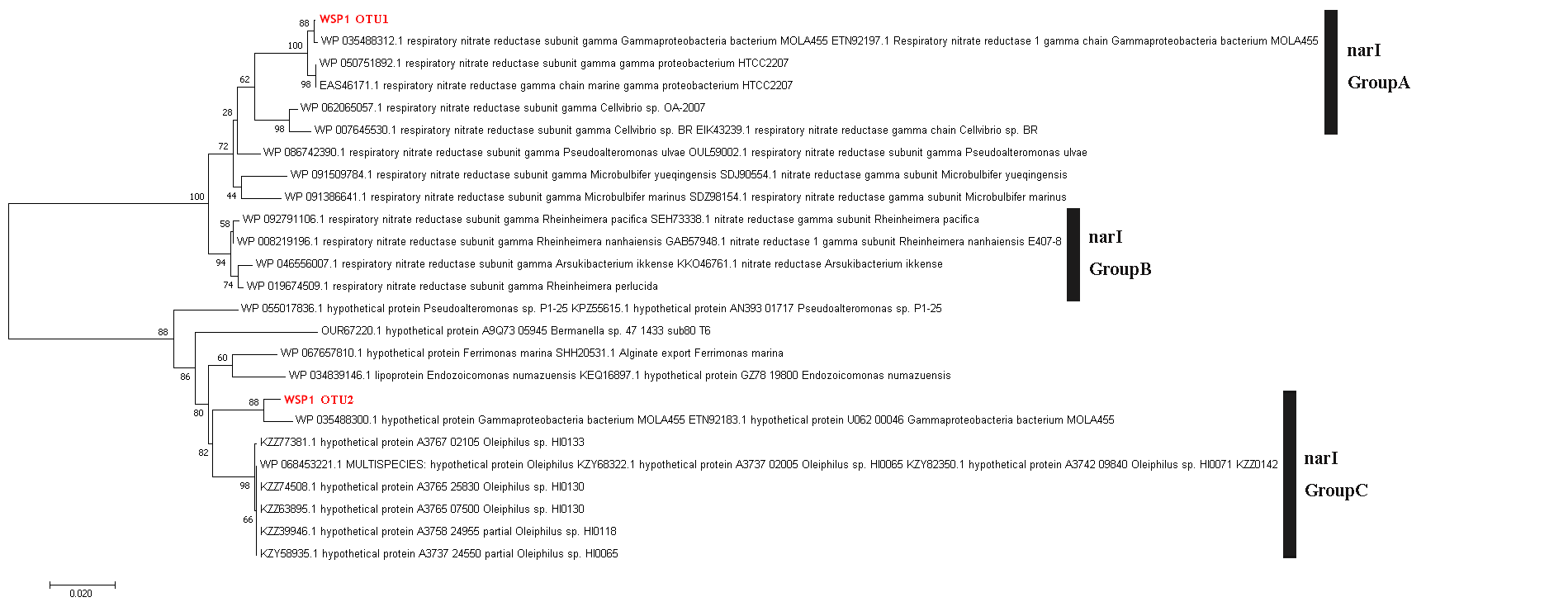
**

**Fig. S10** Phylogenetic tree to show the clustering of the *nirA* gene


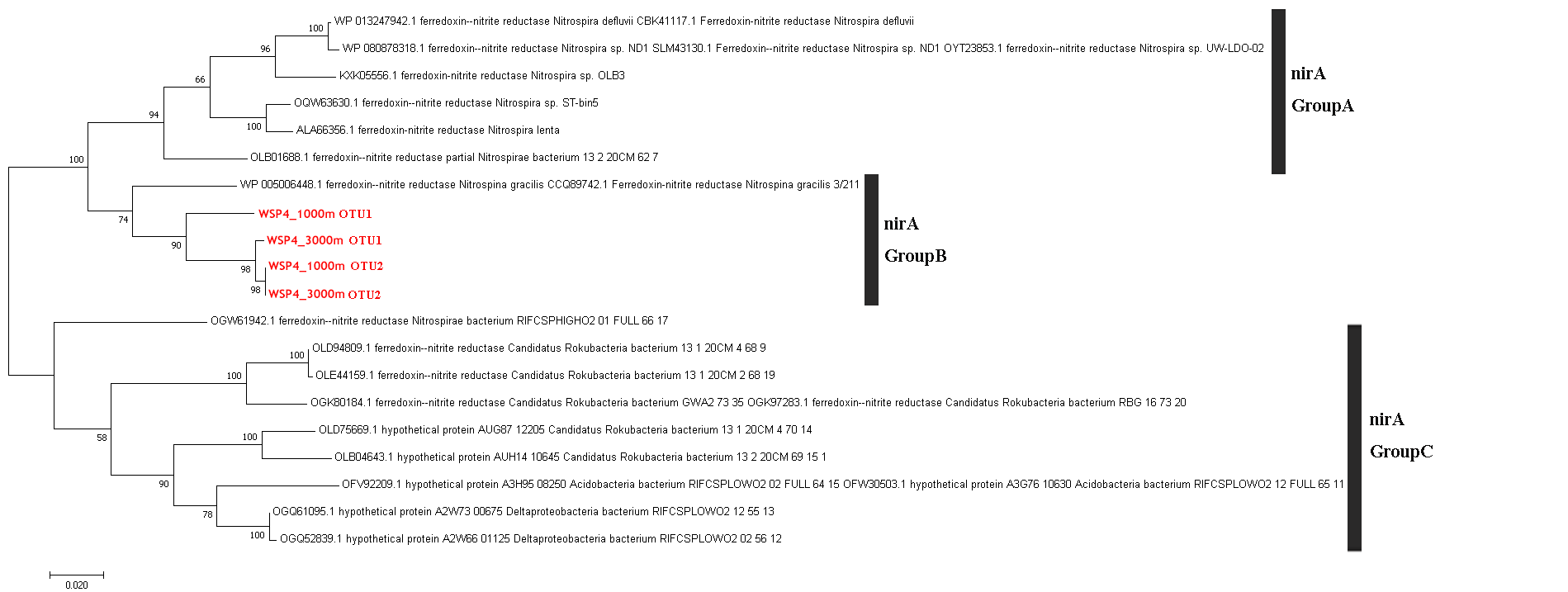


**Fig. S11** Phylogenetic tree to show the clustering of the *nirB* gene


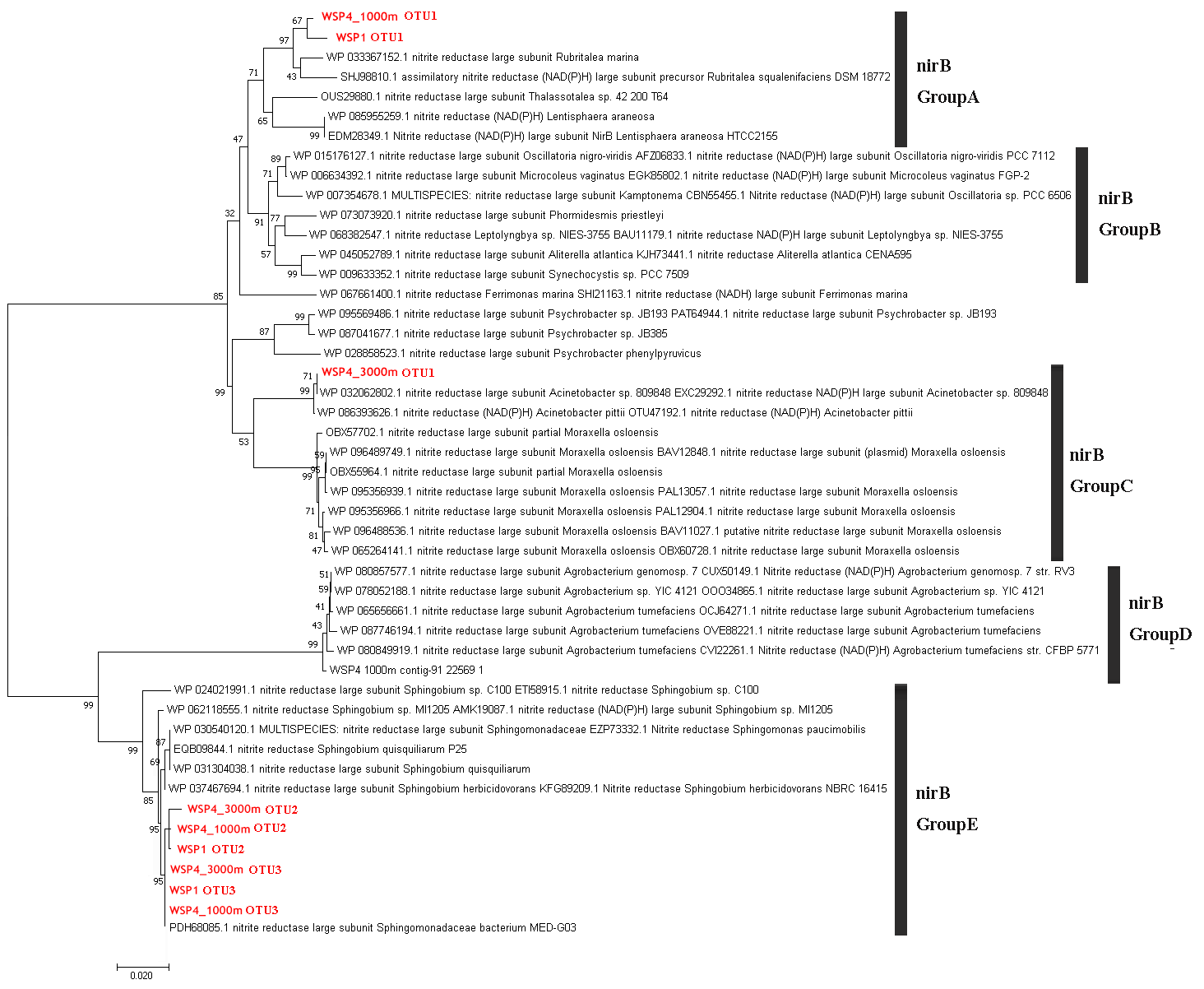


**Fig. S12** Phylogenetic tree to show the clustering of the *nirD* gene


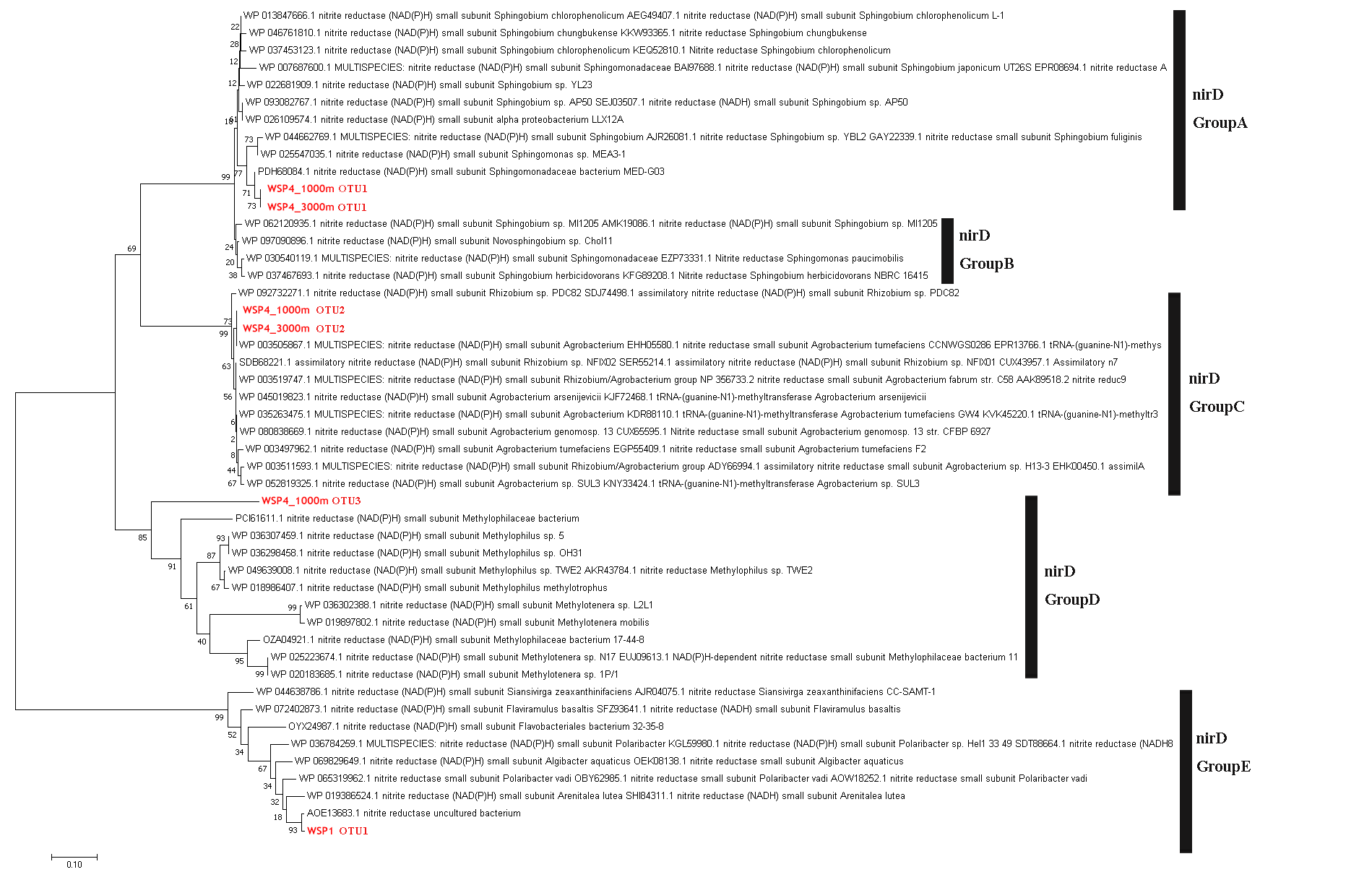


**Fig. S13** Phylogenetic tree to show the clustering of the *nirK* gene


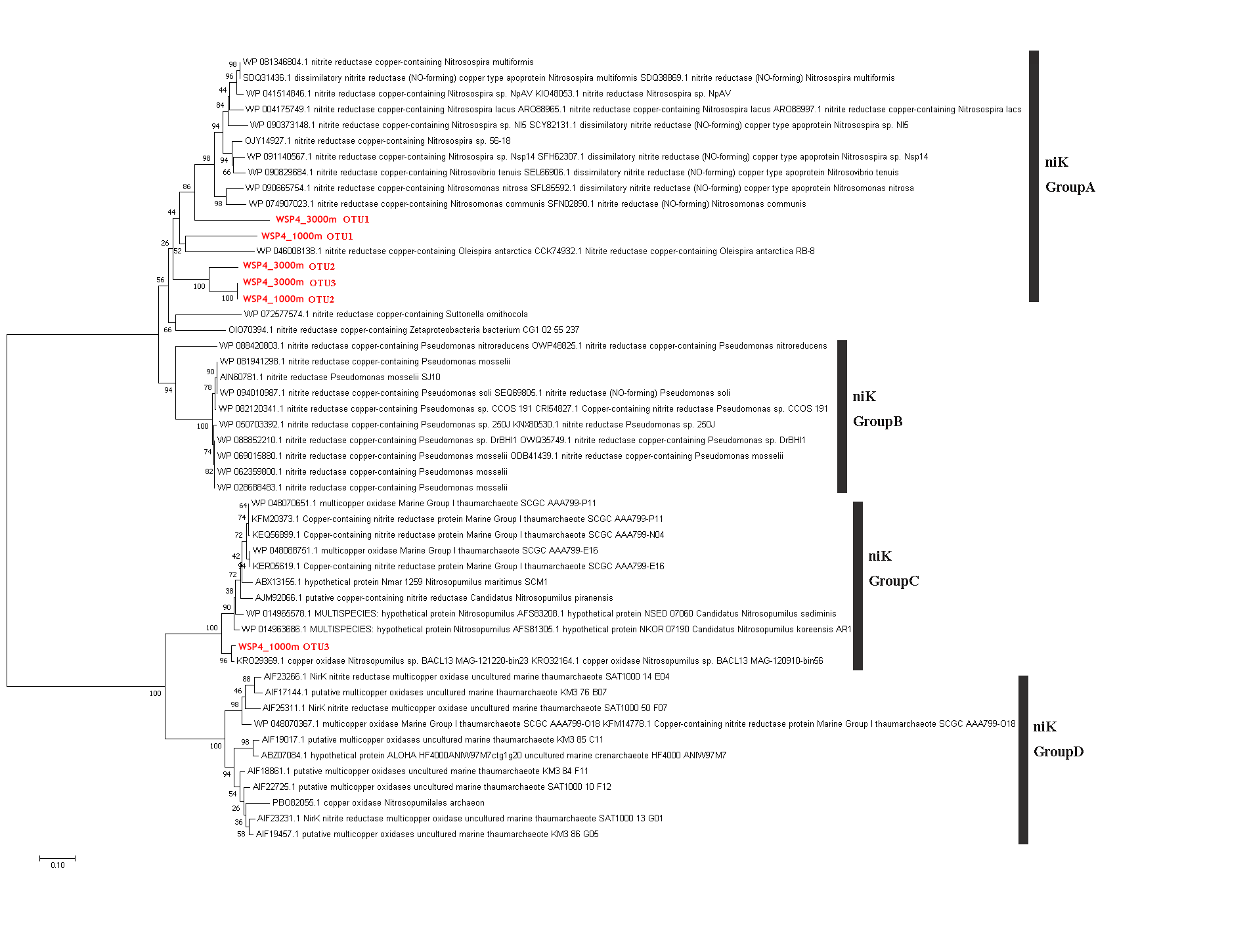


**Fig. S14** Genetic heatmap to show the amount of metabolism of specific amino acids in WSP1 and WSP235. The abundance of each category was log10 scaled.


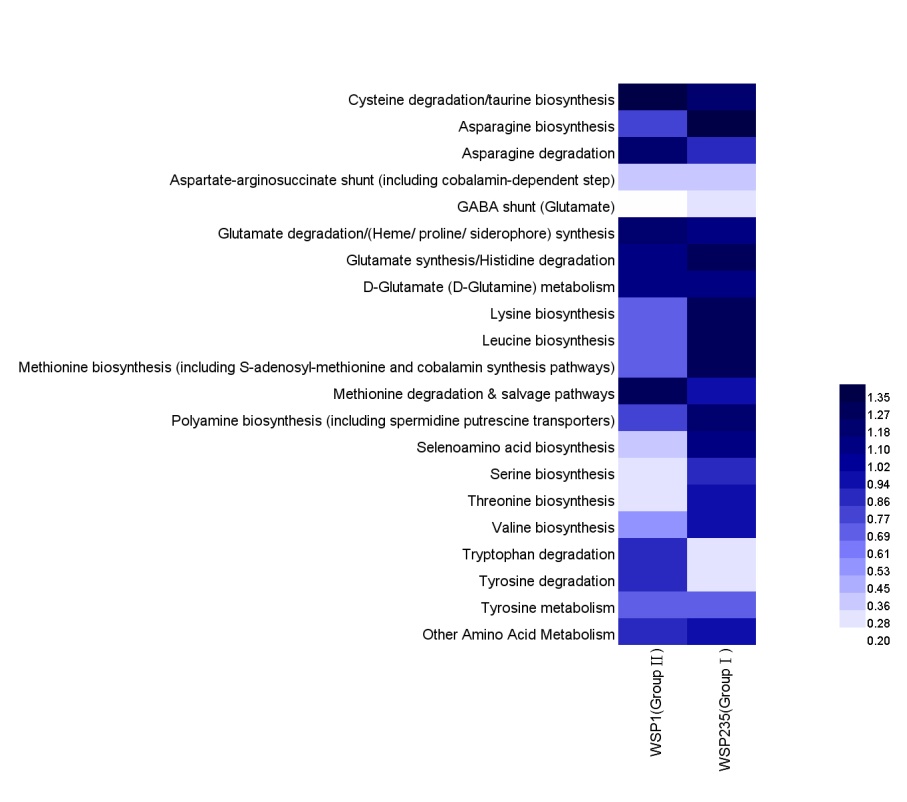

Supplement: Supplementary file 1 [file Data_Sheet_1.DOC]
